# Heat stress causes chromatin accessibility and related gene expression changes in crown tissues of barley (*Hordeum vulgare*)

**DOI:** 10.1007/s11103-024-01509-x

**Published:** 2024-10-22

**Authors:** Agnieszka Kiełbowicz-Matuk, Cezary Smaczniak, Krzysztof Mikołajczak, Anetta Kuczyńska, Xiaocai Xu, Caroline Braeuning, Paweł Krajewski

**Affiliations:** 1 Institute of Plant Genetics, Polish Academy of Sciences, Strzeszyńska 34, Poznań, 60-479 Poland; 2 Department for Plant Cell and Molecular Biology, Institute for Biology, Humboldt-Universität zu Berlin, Rhoda- Erdmann-Haus, Philippstraße 13, 10115 Berlin, Germany; 3 Genomics Platform, Max Delbrück Center for Molecular Medicine in the Helmholtz Association/Berlin Institute of Health, Hannoversche Straße 28, 10115 Berlin, Germany

**Keywords:** Open chromatin regions, Transcriptional regulation, *sdw1*, Crown tissue, High temperature, *Hordeum vulgare*

## Abstract

Plant responses to stress caused by high temperatures involve changes occurring at the molecular, metabolic, and physiological levels. Understanding the mechanisms by which plants recognize signals to activate this response is a prerequisite for identifying key genes and signaling pathways and for obtaining heat-tolerant plants. We demonstrated the first implementation of an assay for transposase-accessible chromatin to identify open chromatin regions (OCRs) in crown tissues of barley using three genotypes carrying different allelic forms of the *sdw1* gene encoding gibberellin 20*-*oxidase subjected to elevated temperatures. In parallel, we performed gene expression analysis, which allowed us to relate changes in chromatin state to changes in transcriptional activity. The obtained data revealed that the hypersensitive chromatin regions within the genes were more repeatable than those outside the gene intervals. We observed that prolonged exposure to high temperatures increased chromatin accessibility. Genes with OCRs in their regulatory regions were involved in stress signaling and tolerance, including calcium-dependent protein kinase, mitogen-activated protein kinase (MAPK3), receptor-like cytoplasmic kinase (RLK), TIFY domain-containing transcriptional regulator, bZIP transcription factor, and regulatory protein NPR1. The effect of genotype on gene expression was not as pronounced as that of temperature. By combining results from the differential analysis of chromatin accessibility and expression profiles, we identified genes with high temperature-induced changes in chromatin accessibility associated with expression alterations. Importantly, our data revealed a relationship between the loss of chromatin accessibility in response to heat and the downregulation of genes related to gibberellin signaling.

**Supplementary Information:**

The online version contains supplementary material available at 10.1007/s11103-024-01509-x.

## Key message

ATAC-seq and RNA-seq in barley revealed temperature-induced changes in chromatin accessibility associated with gene expression alterations, including the downregulation of genes related to gibberellin signaling.

**Supplementary Information:**

The online version contains supplementary material available at 10.1007/s11103-024-01509-x.

## Introduction

High temperatures (HT) can cause serious damage to plants and negatively affect their growth and development. Plant response to stress caused by HT is a complex phenomenon that results from changes occurring at the molecular, metabolic, and physiological levels. HT-induced changes affect protein synthesis, carbohydrates, lipids, hormones, enzymes, and reactive oxygen species, resulting in yield loss by affecting fertility and seed set (Goraya et al. 2017; Moore et al. 2021; Shrestha et al. 2022; Khan et al. 2023). Understanding the mechanisms by which plants perceive and transmit stress signals to activate transcription factors that modulate gene activities associated with the heat stress response is a prerequisite for identifying key genes and signaling pathways to obtain heat-tolerant plants.

Barley (*Hordeum vulgare* L.) is one of the world’s most important cereal crops and is used for feed, malt, and beer production. Productivity and quality are crucial to global food security. Among cereals, barley ranks fourth in terms of grain production worldwide, demonstrating the economic importance of this monocotyledonous crop (Tricase et al. 2018). Similar to other cereals, barley is highly sensitive to HT, leading to poor plant growth and development and low yields (Zhao et al. 2017; Ahmad et al., 2021; Xu et al. 2021; Khan et al. 2023). Prolonged exposure of plants to moderately HTand a few minutes of exposure to extremely HT can cause serious damage, including the denaturation or degradation of proteins, increased fluidity of membrane lipids, loss of membrane integrity, enzyme inactivation, and cell death (Moore et al. 2021). Increasing the average daily temperature by several degrees significantly reduces the grain yield (Lobell et al. 2011). In spring barley genotypes, elevated temperature (24/17°C, day/night) decreased grain yield by 56% (Ingvordsen et al. 2015). In cereals, root and shoot regeneration after exposure to stress is controlled by meristems located in the crowns (first node above the seed), which are specific meristematic tissues involved in the origin of stems, leaves, and roots. Therefore, the ability of plants to survive stress depends on the vitality of their crown tissues (Vítámvás et al. 2015), which remains poorly understood. Furthermore, little is known about the molecular mechanisms underlying heat stress response in barley crown tissues.

The transcription of eukaryotic genes is associated with the formation of open chromatin, which permits the recruitment of transcription factors and other transcriptional regulators. Obtaining data on open chromatin, especially from the large genomes of cereal plants, is highly desirable for the exploration of functional *cis*-regulatory elements that recruit transcription factors to form protein–DNA complexes. To identify open chromatin regions (OCRs), several direct techniques have been introduced, including deoxyribonuclease I sensitive site sequencing (DNase-seq), formaldehyde-assisted isolation of regulatory element sequencing (FAIRE-seq), and assay for transposase-accessible chromatin using sequencing (ATAC-seq) (Zhang et al. 2015; Buenrosto et al., 2015; Bajic et al. 2018; Baum et al. 2020). ATAC-seq utilizes the activity of the Tn5 transposase, which inserts sequencing adapters into accessible chromatin regions. Sequencing can then be used to map regions of increased accessibility as well as to infer regulatory regions and nucleosome positions (Buenrostro et al. 2013, 2015). To date, ATAC-seq technique has been used successfully to identify OCRs in different species, including *Arabidopsis thaliana*, rice (*Oryza sativa*), *Medicago truncatula*, maize (*Zea mays*), wheat (*Triticum aestivum*), sorghum (*Sorghum bicolor*), barley (*Hordeum vulgare*), and tomato (*Solanum lycopersicum*) (Wilkins et al. 2016; Lu et al. 2017; Bajic et al. 2018; Ricci et al. 2019; Lu et al. 2019; Lu et al. 2020; Zhou et al. 2020; Wang et al. 2022). Furthermore, while there are a few data on changes in chromatin accessibility in response to heat stress treatment in rice (Qiu et al. 2023) and tomato (Huang et al. 2023), there is no input in barley, especially in the crown tissue, which is protected under adverse conditions because of its pivotal role in plant development.

In the present study, we applied the ATAC-seq strategy to identify OCRs and to characterize their distribution and organization in the crown tissues of three barley genotypes carrying different allelic forms of the *sdw1* gene encoding gibberellin (GA) 20*-*oxidase (GA20ox) subjected to high temperatures. The choice of plant material reflects the fundamental role of GAs in whole-plant stature formation (Yamaguchi 2008), a process that originates in the crown tissue and is affected by environmental conditions (Zhuang et al. 2019; Yan et al. 2023). We investigated the relationship between changes in chromatin accessibility and differences in gene expression using a parallel RNA-seq assay. Analysis of the crown tissues under different temperature regimes allowed us to identify differences in chromatin accessibility and structure between the analyzed genotypes under heat stress.

## Materials and methods

### Plant material

Plant material (Figure S1) consisted of two-row spring barley (*Hordeum vulgare* L.) cultivar “Bowman” (Bn) and its two near-isogenic lines (NILs), BW827 (B7) and BW828 (B8), which carry the *sdw1.a* and *sdw1.d* mutations, respectively, derived from the “Jotun” and “Valticky” varieties by X-ray treatment (Mikołajczak et al. 2022). BW827 and BW828 were developed by recurrent backcrossing of the mutants to “Bowman” (Druka et al. 2011). Seeds from Bowman (acc. no. NGB20079) and the NILs (BW827, acc. no. NGB22264 and BW828, acc. no. NGB22265) were obtained from the National Small Grains Collection (USDA) and the Nordic Genetic Resource Center (NordGen).

The *sdw1.a* allele was induced by X-ray mutagenesis in a Norwegian six-rowed barley Jotun and has been utilized in the breeding of semi-dwarf feed barley cultivars in Western USA, Canada, and Australia (Jia et al. 2009). The *sdw1.d* allele, considered one of the most crucial for breeding purposes, originated from a mutant selected in the M2 generation of cv. Valticky after X-ray treatment (Hansson et al. 2024). This mutant, officially named cv. Diamant, was introduced in Czechoslovakia in 1965, and subsequently this allele has played a key role in the development of over 150 new malting barley cultivars across Europe (Kuczyńska et al. 2013). Comparative genomic analysis revealed that the *sdw1* gene in barley is located in the syntenic region of the rice green revolution semi-dwarf gene *sd1*, encoding a gibberellin 20-oxidase enzyme (Xu et al. 2017). The *sdw1.a* allele resulted from a total deletion of the *HvGA20ox2* gene and partial or total loss of function of this gene could be compensated by enhanced expression of its homolog *HvGA20ox1* and *HvGA20ox3.* Noteworthy, Mikołajczak et al. (2022) demonstrated that the deletion around the *sdw1.a* locus amounted to 0.555 Mb including 13 genes at least. In turn, the *sdw1.d* allele is caused by a 7-bp deletion in exon1, which resulted in a coding frame shift and premature translation termination. As there is an internal start codon ATG, the *sdw1.d* allele may lead to a truncated protein with a conserved domain of the 2-oxoglutarate (2OG) and Fe(II)-dependent oxygenase superfamily (Cheng et al. 2023). Thus, the *sdw1.d* allele still maintains partial function of GA 20-oxidase.

### Experimental design

Plants were grown in pots (H-LSR 4.5 L; 21 cm in diameter and 20 cm in height) filled with a mixture of clay soil and peat (3:1, w/w) under controlled conditions with 16-h photoperiod (16 h light / 8 h dark), 60% humidity and PAR irradiance of 234 µmol m^− 2^ s^− 1^ (Apollo 8 LED Grow Light). Each pot was weighed daily to maintain the soil moisture above 70% of the field water capacity. The number of pots was set to provide material for all the studies. Eight seeds were sown per pot and, after germination, the number of plants was limited to five. Two temperature variants were used: (i) control temperature (C) 16/8°C (day/night) from sowing to the end of tillering, followed by 20/12°C (day/night) to maturity; (ii) high temperature (HT) 28 °C from sowing to the end of tillering, followed by 20/12°C (day/night) as in the control. Following these treatments, barley crown tissues (Figure S1; Figure 4 in Mikołajczak et al. 2022) were sampled, frozen immediately in liquid nitrogen, and stored at -80 °C for RNA-seq analysis or immediately cross-linked in 1% formaldehyde under vacuum infiltration for 30 min for nuclei extraction. Cross-linking was stopped by adding glycine to a final concentration of 0.125 M and vacuum infiltration was extended for 5 min. Subsequently, the material was stored at -80 °C until use.

### RNA-seq

Analysis of gene expression in the crown tissues of the Bn cultivar and B7 and B8 NIL lines under control and heat-treated conditions at two time points: 1d – tillering stage, and 10d – 10 days after 1d was carried out using the mRNA-seq method. The mRNA-seq analysis was performed using three biological replicates. Each replicate consisted of crown samples collected from three plants per pot. Total cellular RNA was extracted using TRI Reagent^®^ RT (Molecular Research Center, Inc., Cincinnati, OH, USA) according to the manufacturer’s protocol and treated with DNase I during RNA purification. The quality and quantity of RNA were verified using a NanoDrop 2000 spectrophotometer (Thermo Fisher Scientific) using the following criteria: 2.0 for 260/280 and 260/230 ratios. RNA integrity number (RIN) of samples sufficient for sequencing (≥ 8) was confirmed using an Experion™ electrophoresis station (Bio-Rad Laboratories, Hercules, CA, USA). cDNA library construction (TruSeq stranded mRNA) and sequencing were conducted by Macrogen Inc. (Seoul, Republic of Korea) using an Illumina platform with a 2 × 150 bp PE configuration.

### Preparation of crude nuclei

The procedure involving the isolation of pure and undamaged barley nuclei constitutes a crucial phase in plant nucleus ATAC-seq methodology. This process encompasses the delicate yet highly efficient extraction of plant nuclei from swift snap-frozen barley tissue specimens. Samples for nuclear isolation were collected in the main experiment of the project using the same scheme as for the mRNA-seq gene expression analysis, but in two replicates. Purified nuclei were isolated at 4 °C from barley cross-linked crown tissues according to a modified method of Moreno-Romero et al. (2017). Then, 3–4 crown samples were crushed gently into small pieces in liquid nitrogen using a mortar and a pestle (approximately 10 times) to obtain the big chunks and then transferred to GentleMACS M tubes filled with 5 mL of Honda buffer (2.5% w/v Ficoll 400, 5% dextran T40, 0.4 M sucrose, 25 mM Tris-HCl, pH 7.4, 10 mM MgCl_2_) supplemented with 10% Triton X-100, 1 M DTT and 1 tablet / 50 mL cOmplete Protease Inhibitor Cocktail (Roche). This buffer composition allows for the efficient lysis of cell membranes while maintaining unaffected nuclear membranes. To disrupt the tissue and release the nuclei, the samples were homogenized in GentleMACS M tubes using a MACS Dissociator with a plant-specific program (10.1111/tpj.15458). Homogenate was filtered through 70-µm nylon mesh cell strainer and pelleted by centrifugation (4000 *g*) at 4 °C for 6 min. The pellet was resuspended in 5 mL 1x PBS buffer (Invitrogen) with gentle mixing and centrifuged at 1,000 *g* at 4 °C for 6 min. Finally, the pellet containing nuclei was resuspended again in 250 µL 1x PBS and filtered through 35-µm cap mesh into a sorting tube to remove large debris. DAPI (100 µg/mL) was added to a final concentration of 2–3 µM DAPI for staining. Intact nuclei were discriminated and sorted from broken nuclei and small debris by the DAPI staining with a flow cytometer (BD FACSAria III). For subsequent experiments, 150,000 DAPI positive events were sorted into a 1,5mL Eppendorf tube containing 20 µl of collection buffer (4% BSA in 1x PBS). Sorted nuclei were counted and checked for integrity using a Countess II FL Automated Cell Counter (Thermo Fisher Scientific).

### Tagmentation with Tn5 and library preparation

Freshly sorted nuclei destined for use in ATAC-seq were spun down at 1500 *g* for 10 min at 4 °C and stored on ice prior to the transposase integration reaction. Tagmentation was performed in 2x Tn5 buffer (Illumina) using 50k nuclei and hyperactive transposase Tn5 (Illumina). Reactions were performed at 37 °C for 30 min, with gentle shaking at 1,000 rpm and then stopped at 55 °C for 10 min in one volume of STOP buffer (50 mM Tris-HCl, pH 8.0; 100 mM NaCl, 0,1% SDS; 100 mM EDTA, pH 8.0). To obtain nucleosome-free DNA substrate the samples were subjected to overnight incubation with 2.5 µL Proteinase K (20 mg/ml) at 65 °C followed by adding 10% Triton X-100 (final concentration 1%) to quench SDS. DNA fragments were purified using the ZymoBIOMICS MagBead DNA Kit (Zymo Research), eluted in 20 µL of elution buffer, and then amplified using Next High Fidelity PCR Mix (NEB) and specific primers for 9–19 total PCR cycles. The amplified ATAC-seq libraries were quantified via qPCR using the NEBNext Library Quantification Kit (NEB), purified using AMPure XP beads (Beckman Coulter), and analyzed for library concentration using Qubit and size distribution using TapeStation (Agilent). Sequencing of the libraries was performed by Novogene using the Illumina platform in 2 × 150 bp PE mode.

### RNA-seq data processing and analysis

After removing adapter-related sequences and quality trimming using AdapterRemoval ver 2.1.7 (RRID: SCR_011834, https://github.com/MikkelSchubert/adapterremoval, Schubert et al. 2016) with parameters: min quality 20, min length 50, mRNA-seq reads were mapped using TopHat ver. 2.1.1 (RRID: SCR_013035, http://ccb.jhu.edu/software/tophat/index.shtml, Kim et al. 2013) in MorexV3 pseudomolecules (Monat et al. 2019; Ensembl Plants rel. 55; RRID: SCR_008680, http://plants.ensembl.org) treated as the reference genome; abbreviated gene identifiers used in the text do not include the “HORVU.MOREX.r3.” prefix. Reads aligned to annotated transcripts were counted using the featureCounts function in Bioconductor (RRID: SCR_006442, http://www.bioconductor.org/) in R 3.6.1 (Rsubread library, Liao et al. 2019), and the resulting data were subjected to differential expression analysis in Deseq2 ver. 1.22.2 (RRID: SCR_015687, https://bioconductor.org/packages/release/bioc/html/DESeq2.html, Love et al. 2014). DEGs (genes differing in expression between two experimental variants) were found among the genes characterized by a mean expression of at least five units (estimated in Deseq2), with the following conditions: BH-corrected p-value < 0.05,|log2(FC)| > 2. GO term enrichment analysis was performed using the hypergeometric test, with the computation of family-wise error rates (FWER < 0.01) using the GOfuncR library ver. 1.18.0 in Bioconductor (Grote 2020).

### ATAC-seq data processing and analysis

The next generation sequencing datasets were subjected to quality processing involving the removal of adapters, duplicate sequences, poly G sequences, missing sequences (N), and low-quality sequences (< Q15) using the fastp tool (RRID: SCR_016962, https://github.com/OpenGene/fastp, Chen et al. 2018). Reads remaining in the analysis were mapped to the barley MorexV3 pseudomolecule reference sequence using Bowtie2 software (parameter -score-min L,0,-0.15) (RRID: SCR_016368, http://bowtie-bio.sourceforge.net/bowtie2, Langmead and Salzberg 2012). Reads mapped to the mitochondrial and chloroplast sequences were removed from the mapped datasets. The resulting data were used to identify regions of accessible chromatin (enriched in mapped DNA reads) using the method implemented in Macs2 software with parameters (nomodel, q-value < 0.05) (RRID: SCR_013291, https://github.com/macs3-project/MACS, Zhang et al. 2008). Further data processing (data subsampling, construction of “open chromatin regions”) is described below in Results.

### Statistical analyses, visualization, and gene annotation

Statistical analyses of the obtained data and visualization of the results were performed using Genstat 19 (RRID: SCR_014595, http://www.vsni.co.uk/products/genstat/; VSN Int., 2017). For visual exploration of genomic data, the Integrative Genomics Viewer tool was used (RRID: SCR_011793, http://www.broadinstitute.org/igv/). Venn diagrams were built using the “venn” package in R. Identified genes were annotated on the MorexV3 pseudomolecules in Ensembl Plants. Annotation of barley genes with respect to the KEGG (RRID: SCR_012773, http://www.kegg.jp) and Plant Reactome (https://www.plantreactome.gramene.org/) pathways was performed using the OmicsBox (RRID: SCR_023676, https://www.biobam.com). The GOfuncR package in R was used to perform GO term enrichment (overrepresentation) analysis.

## Results

### Isolation of nuclei

**Fig. 1:**
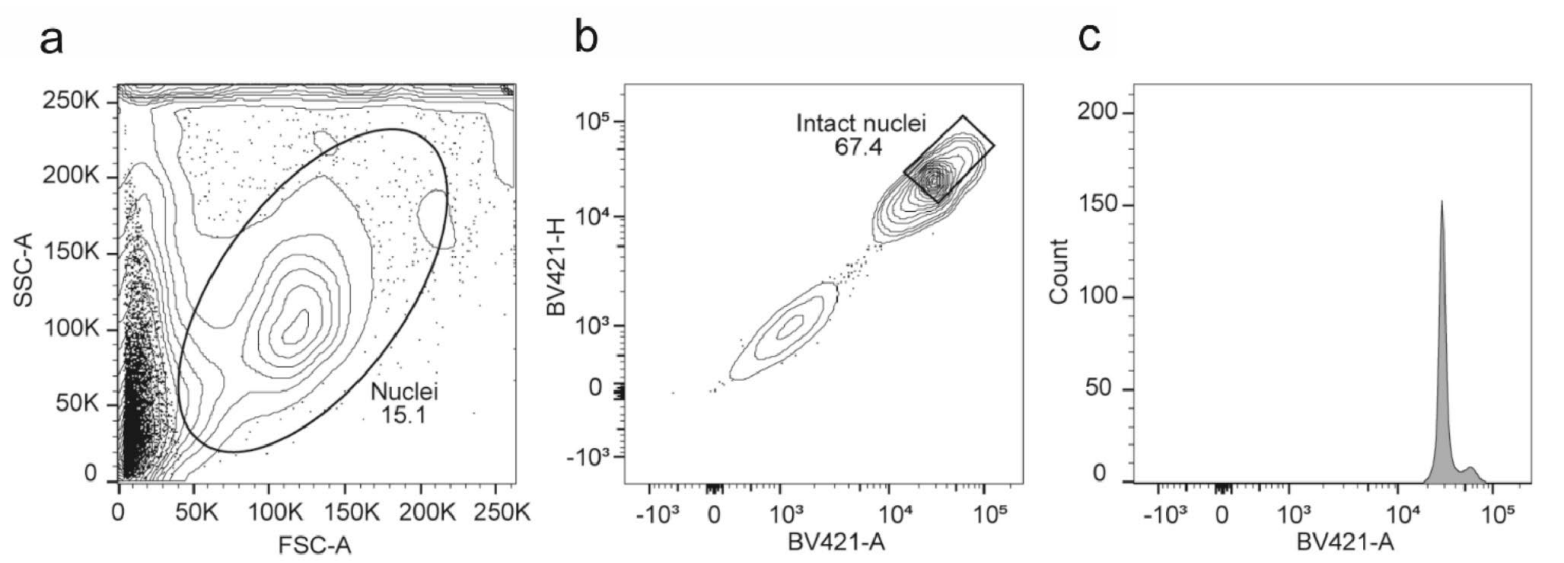
FACS gating strategy to sort intact barley nuclei based on DAPI staining. **(a)** First debris and aggregates were excluded in a FSC-A/SSC-A plot. **(b)** Subsequently intact nuclei and doublets were excluded and intact nuclei gated. **(c)** The histogram plot shows the sharp DAPI profile of intact high quality nuclei

**Fig. 2:**
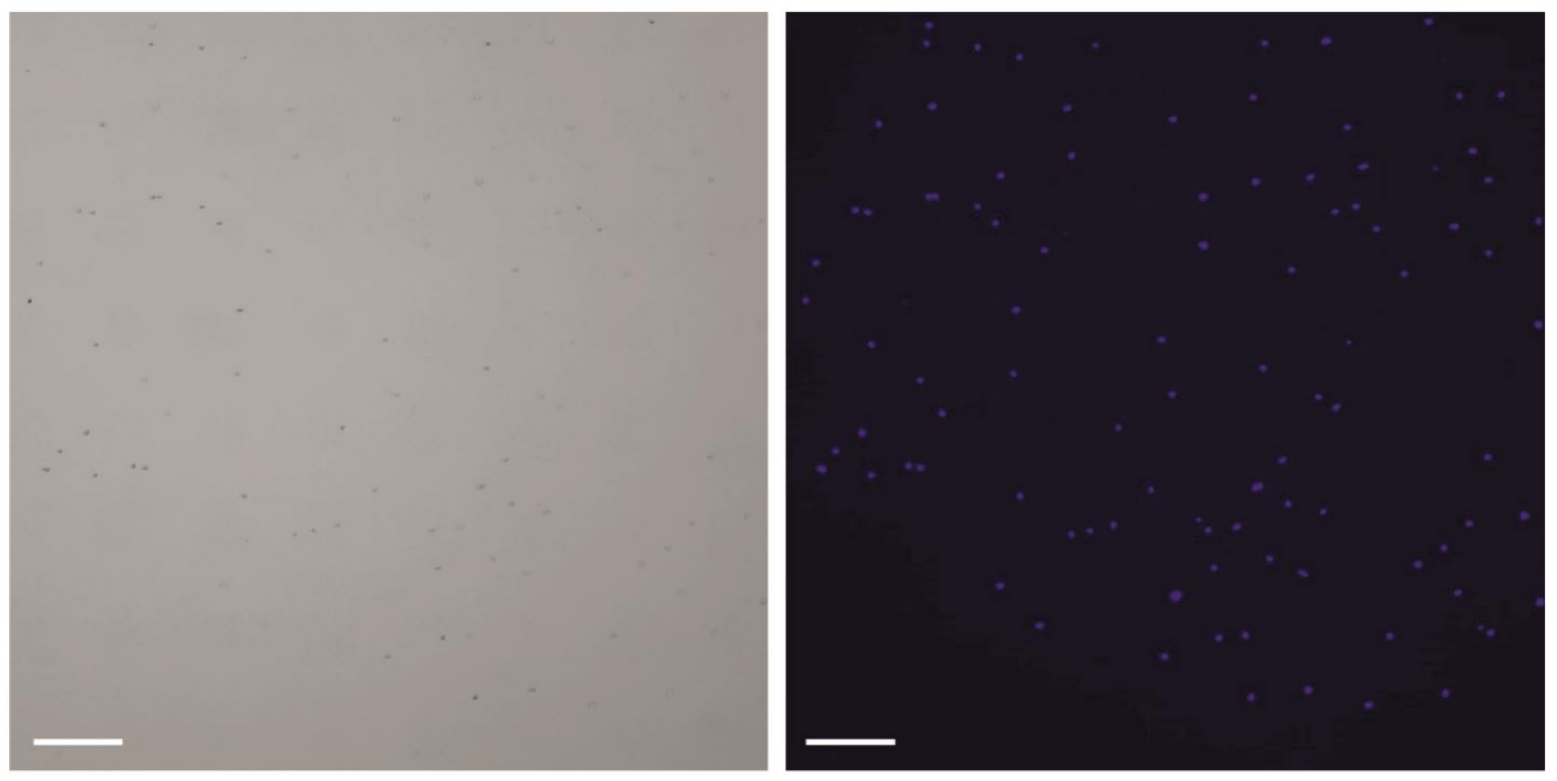
Microscopic analysis of FACS-sorted barley nuclei. Left: bright field; right: DAPI staining. Scale bar is 200 μm

Intact nuclei from the barley specimens were isolated from the residual cellular debris via fluorescence-activated cell sorting (FACS) with an appropriate gating strategy (Fig. 1). This separation between the nuclei and debris was verified by microscopic examination of DAPI-stained barley nuclei (Fig. 2). Intact nuclei were quantified, and 50,000 nuclei were used for bulk ATAC-seq experiments.

### Construction of merged sets of OCRs

The number of ATAC-seq read pairs (PE reads) obtained from samples varied from 12.4 to 181.8 M, the number of those not mapped in nonchromosomal sequences – from 10.4 to 163.6 M, and the number of single reads mapped in chromosomes 1–7 H – from 7.7 to 257.4 M (Table S1a). The mapping rate was the smallest for data on (B7, HT, 1d) – 4.12% on average for two replicates – and the largest for (Bn, HT, 10d) – 71.64%.

For replicates in which the number of mapped reads was larger than 10 M, the mapped reads were subsampled to 10 M (Table S1a). The number of peaks called in Macs2, hereinafter referred to as OCRs, varied from 136 for sample (Bn, C, 10d, rep 1) to 24,126 for sample (B7, HT, 10d, rep 2). The reproducibility of OCRs, measured as the percentage of OCRs from the smaller set intersecting with OCRs from the larger set, varied from 7.21% (B8, C, 10d) to 90.23% (Bn, C, 10d).

**Fig. 3:**
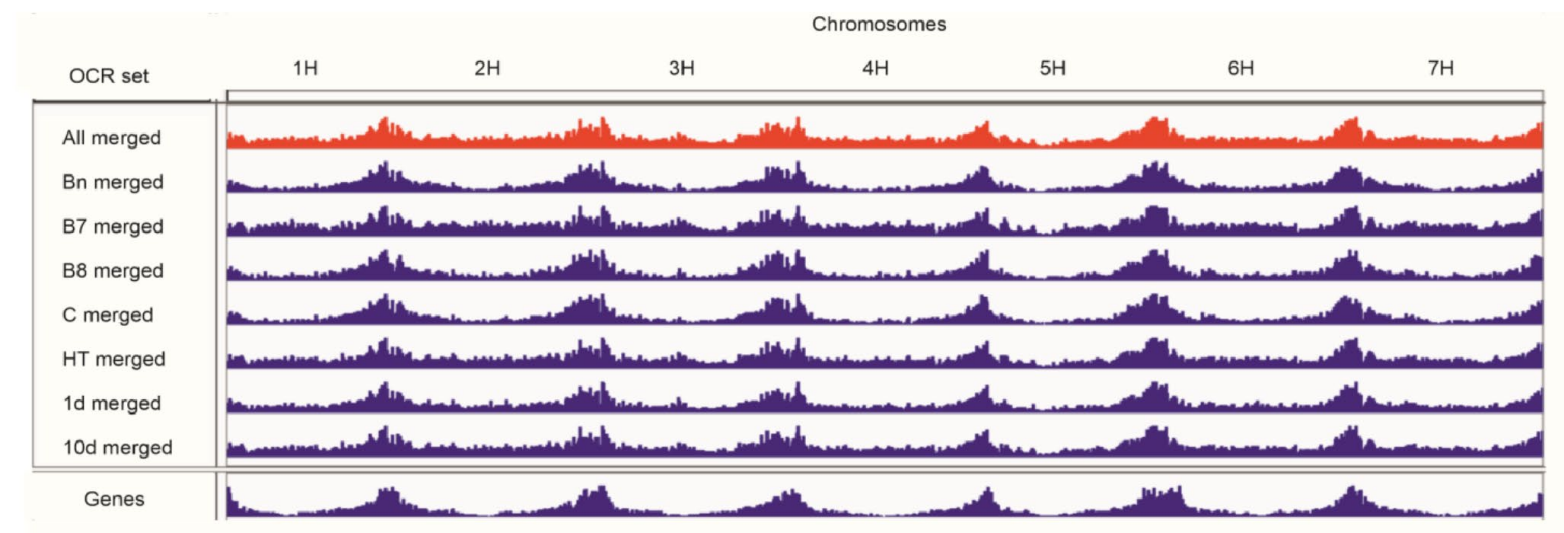
Open chromatin regions in sets merged for experimental variants in seven chromosomes of barley. Red – merged from all replicates, blue – merged within variants of experimental factors, see Table 1

Sets of OCRs corresponding to genotypes, time points, and temperature regimes combinations were made by finding the sets of peaks declared by Macs2 appearing in at least one replicate, in appropriate variant, and were called “marginal sets” Bn, B7, B8, 1d, 10d, C and HT; similar sets of OCRs were constructed for combinations of genotypes and time points (Table 1). The distribution of OCRs across chromosomes is illustrated in Fig. 3.

**Table 1:** Numbers of OCRs obtained by merging peaks over replicates within experimental variants

Experimental variant - genotype, time point	Number of merged OCRs		
	Temperature regime		Marginal set
	Control (C)	High (HT)	
Bn	17,754	27,918	30,807
B7	12,452	41,315	49,906
B8	15,657	5424	19,678
1d	21,406	18,657	37,615
10d	14,857	58,968	60,828
Marginal set	24,901	65,140	70,101

### Characteristics of OCR sets and accessible genes

**Fig. 4:**
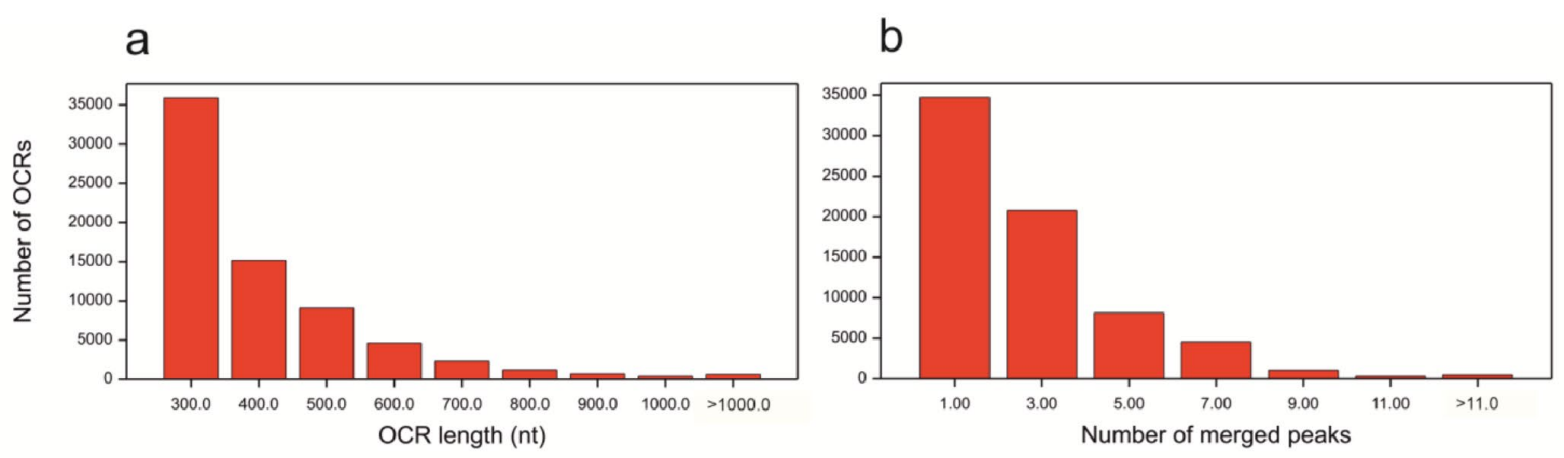
Characteristics of open chromatin regions. **(a)** Distribution of region length. **(b)** Distribution of the number of peaks merged into one region. The groups are labeled by upper limits of the variables, except for the last one

The set of OCRs merged from all 24 replicates contained 70,101 elements (Table 1). The minimum and maximum lengths of the OCRs were 200 nt and 3346 nt, respectively, and the maximum number of Macs2 peaks that merged into one marginal OCR was 54 (Fig. 4). Out of all the OCRs, 21,521 intersected with 16,870 promoter-gene intervals (intervals consisting of promoter regions and gene loci, defined as [TSS-5 kb, TTS + 1 kb]), in the majority of cases (15,767 OCRs, 14,363 genes) with the promoter region [TSS-5 kb, TSS]. OCRs merged from a low number of peaks, thus occurring in a small number of replicates, intersected with promoter-gene intervals in 24.76% of cases, which was significantly less than approximately 40% for OCRs merged from more peaks, whereas OCRs merged from a low number of peaks intersected with promoters in 69.19% of cases, which was significantly less than approximately 85% for OCRs merged from more peaks (Figure S2).

**Fig. 5:**
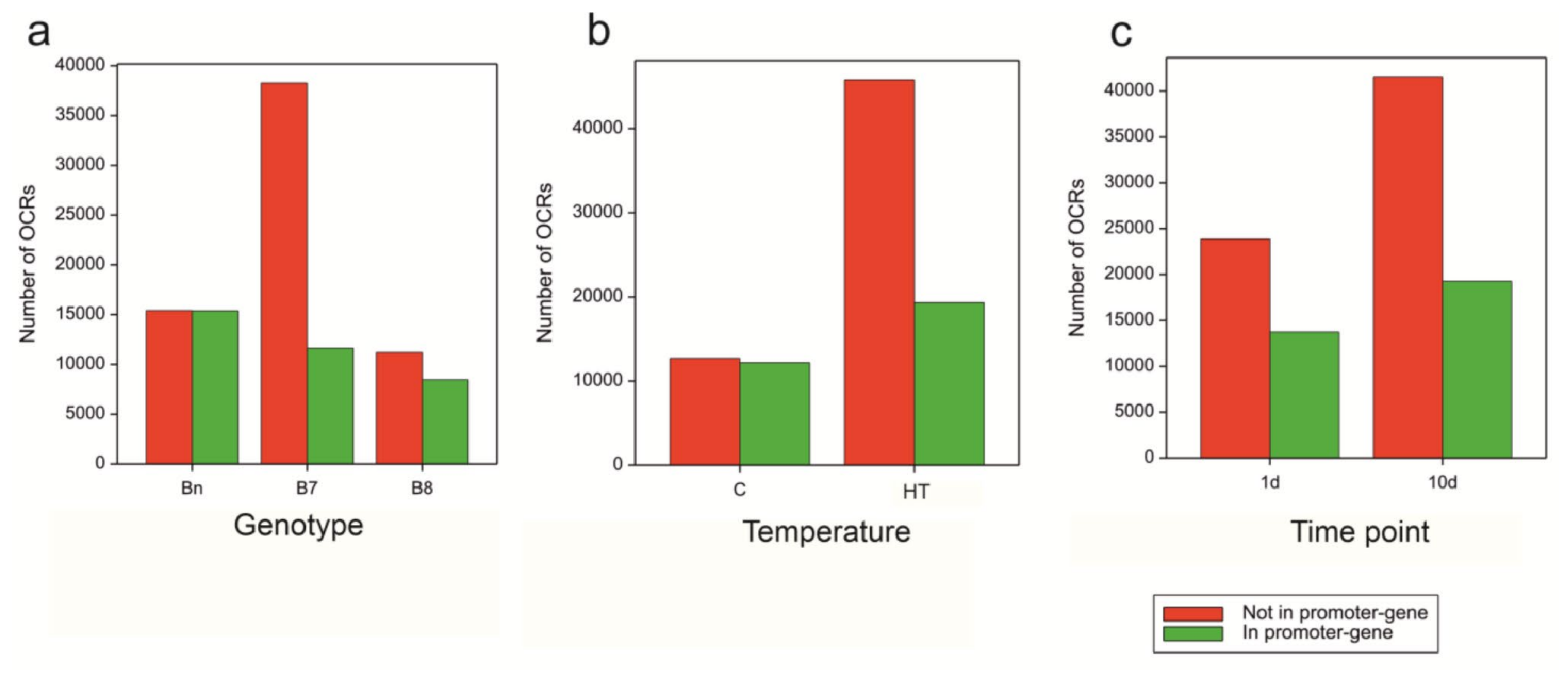
Proportions of OCRs intersecting with promoter-gene intervals, in marginal sets of OCRs for levels of factors: **(a)** Genotype, **(b)** Temperature, **(c)** Time point

The number of OCRs in the marginal sets constructed for the three genotypes varied from 19,678 for B8 to 49,906 for B7 (Table 1). The sets constructed for optimum and high-temperature treatments contained 24,901 and 65,140 OCRs, respectively, while the sets constructed for time points 1d and 10d contained 37,615 and 60,828 OCRs, respectively (Table 1). They were unbalanced, however, the differences between factors levels were caused mainly by the presence of numerous OCRs located outside of promoter-gene intervals. This was visible in particular for genotype B7 and high temperature HT (Fig. 5). The highest number of OCRs was detected at 10d at high temperature (58,968).

The number of accessible genes (i.e., those with OCRs within the promoter-gene interval; for detailed data, see Table S2) for the experimental variants is shown in Table 2. These were approximately proportional to the number of OCRs in the marginal sets, as shown in Table 1. More genes were reported for Bn than for B7 or B8, more at 10 days after tillering (10d) than at tillering (1d), and more under high temperature (HT) than under control temperature (C), with approximately 1/3 of the genes being specific for the largest sets (Figure S3). The largest number of accessible genes was detected at 10d at HT (15,138).

**Table 2:** Numbers of genes with OCRs for experimental variants

Experimental variant - genotype, time point	Number of genes		
	Control temperature (C)	High temperature (HT)	Marginal set
Bn	9174	12,789	13,800
B7	5994	6937	10,252
B8	7476	1331	8093
1d	10,385	3710	12,160
10d	6973	15,138	15,567
Marginal set	11,373	15,561	16,870

The GO annotations of the genes belonging to the constructed sets are reported in Table S3. Representative GO terms that were highly overrepresented in the annotation of the set of all genes with OCRs are shown schematically in Figure S5.

### Differences between experimental variants with respect to gene ontology (GO) terms overrepresented in sets of accessible genes

GO enrichment analysis was used to test the over-representation of gene ontology terms associated with the molecular activity of individual gene products, sites where gene products are active, and the pathways and larger processes to which the activity of that gene product contributes. We analyzed the differences among the three barley genotypes, two temperature regimes, and two time points (Table S3, Figure S4).

We found that 57 GO terms were overrepresented for all barley genotypes: Bn, B7 and B8 (Figure S4a). Moreover, the set of over-represented terms was substantially larger for the Bn variety than for the B7 and B8 NILs (Table S3). Among them were terms corresponding to the regulation of growth, developmental growth, reproductive system development, lipid biosynthesis, and metabolic processes.

When comparing gene sets at 1d and 10d, we revealed that the sets of GO terms overrepresented for gene sets corresponding to different time points were balanced in number (Figure S4b, Table S3), with the majority of terms being common for two time points.

To explore the annotation of genes related to chromatin opening under the two temperature regimes, we performed GO enrichment analysis for gene sets associated with control and high temperatures. We found that the sets of GO terms that were overrepresented for gene sets corresponding to different temperature treatments were also balanced (Figure S4c). Our data showed enrichment of genes related to plant development as well as metabolic process under both treatments, suggesting that most of the accessible chromatin corresponded to “housekeeping” genes that are actively transcribed for proper plant growth and development. However, we found that 40 over-represented GO terms were specific to high temperatures; they included terms corresponding to RNA pathways, protein phosphorylation, regulation of signaling, and regulation of signal transduction (Table S3). These results demonstrate notable variations in chromatin accessibility, especially under different temperature regimes, which may influence changes in the expression of stress-related genes.

### Effect of prolonged temperature on gene accessibility

Unlike the marginal sets described above, the over-representation of GO terms for the sets of genes corresponding to the two temperature regimes within genotypes and time points could not be compared because of an imbalance in the number of genes (Table 2). However, we focused on the largest set of genes with OCRs obtained for (10d, HT), which contained 15,138 genes. We found the most interesting genes associated with the regulation of the stress response, among which 24 were assigned the putative biological function related to stress signaling and tolerance e.g. calcium-dependent protein kinase 29 (2HG0188200), kinase MAPK3 (4HG0385780), receptor-like cytoplasmic kinase (2HG0185050), TIFY domain-containing transcriptional regulator (4HG0405230), bZIP transcription factor (3HG0299650), and regulatory protein non-expresser of pathogenesis-related protein 1 (NPR1) (3HG0236240).

### Gene expression levels

RNA-seq protocol provided 36 data sets, with 17.9–26.0 M read pairs per sample; the mapping rate amounted to 69.4–86.3% (Table S1b). The analysis of gene expression data involved testing the significance of the mean effects of factors (Table 3) and temperature effects separately for the three genotypes and two time points (Table 4). The numbers of differentially expressed genes (DEGs) that were specific and shared for particular contrasts are shown in Figure S6. Very few DEGs were found in the comparisons between genotypes and time points. Most DEGs for HT v. C were found at the time point 1d.

**Table 3:** DEGs in the comparisons of genotypes, time points and temperature regimes

Regulation of DEGs	Contrast of mean expression			
	B7 v. Bn	B8 v. Bn	10d v. 1d	HT v. C
Down	19	16	16	687
Up	6	16	16	336
Total	25	32	32	1023

**Table 4:** DEGs in the comparisons of temperature regimes for three genotypes and two time points

Regulation of DEGs	Contrast HT v. C for				
	genotypes			time points	
	Bn	B7	B8	1d	10d
Down	362	972	605	2170	526
Up	259	471	241	1126	528
Total	621	1443	846	3296	1054

GO overrepresentation analysis for the sets of DEGs is summarized in Table S4 and Figure S7. It shows that no GO terms were overrepresented in the set of DEGs for comparison of B7 v. Bn, and just one for the comparison B8 v. Bn (“cell surface receptor signaling pathway”, GO:0007166) (Figure S7a). No common GO terms were found in the comparison of HT vs. C for the three genotypes. One GO term, “extracellular region” (GO:0005576), was common to Bn and B7.

### Chromatin accessibility versus gene expression

**Fig. 6:**
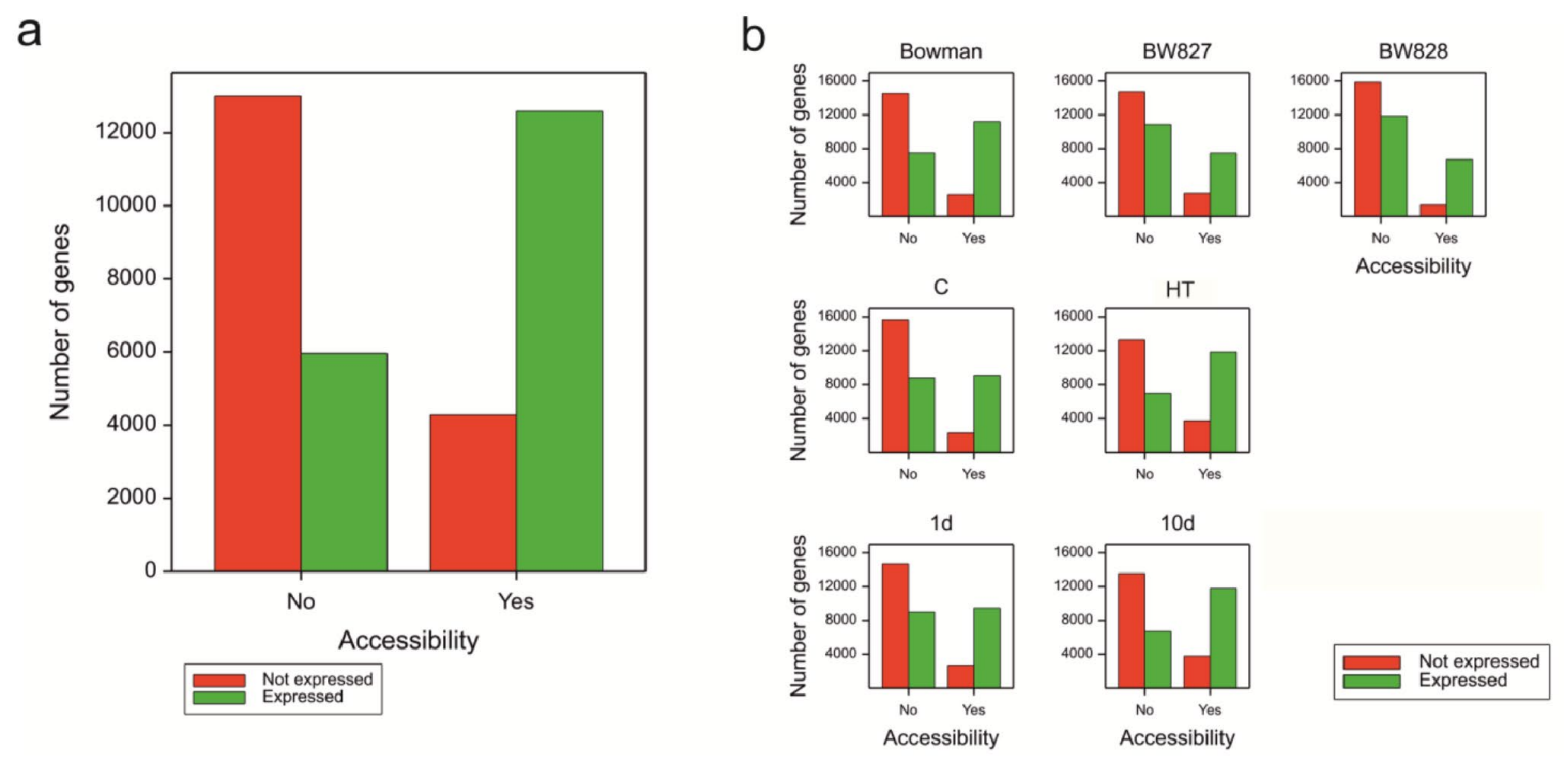
**(a)** Fractions of not expressed and expressed genes among those not having and having OCR in at least one replicate. **(b)** Fractions of not expressed and expressed genes among those not having and having OCR for experimental variants

To evaluate the relationship between changes in chromatin accessibility and gene expression, integrative ATAC-seq and RNA-seq data analyses were performed. Genes with an OCR in at least one replicate (out of 24) were more likely to be expressed (i.e., characterized by a mean FPKM of at least 0.5) in the experiment (Fig. 6a). The same rule was observed for all sets of genes with OCRs from the marginal sets of genotypes, temperature regimes, and time points (Fig. 6b).

**Fig. 7:**
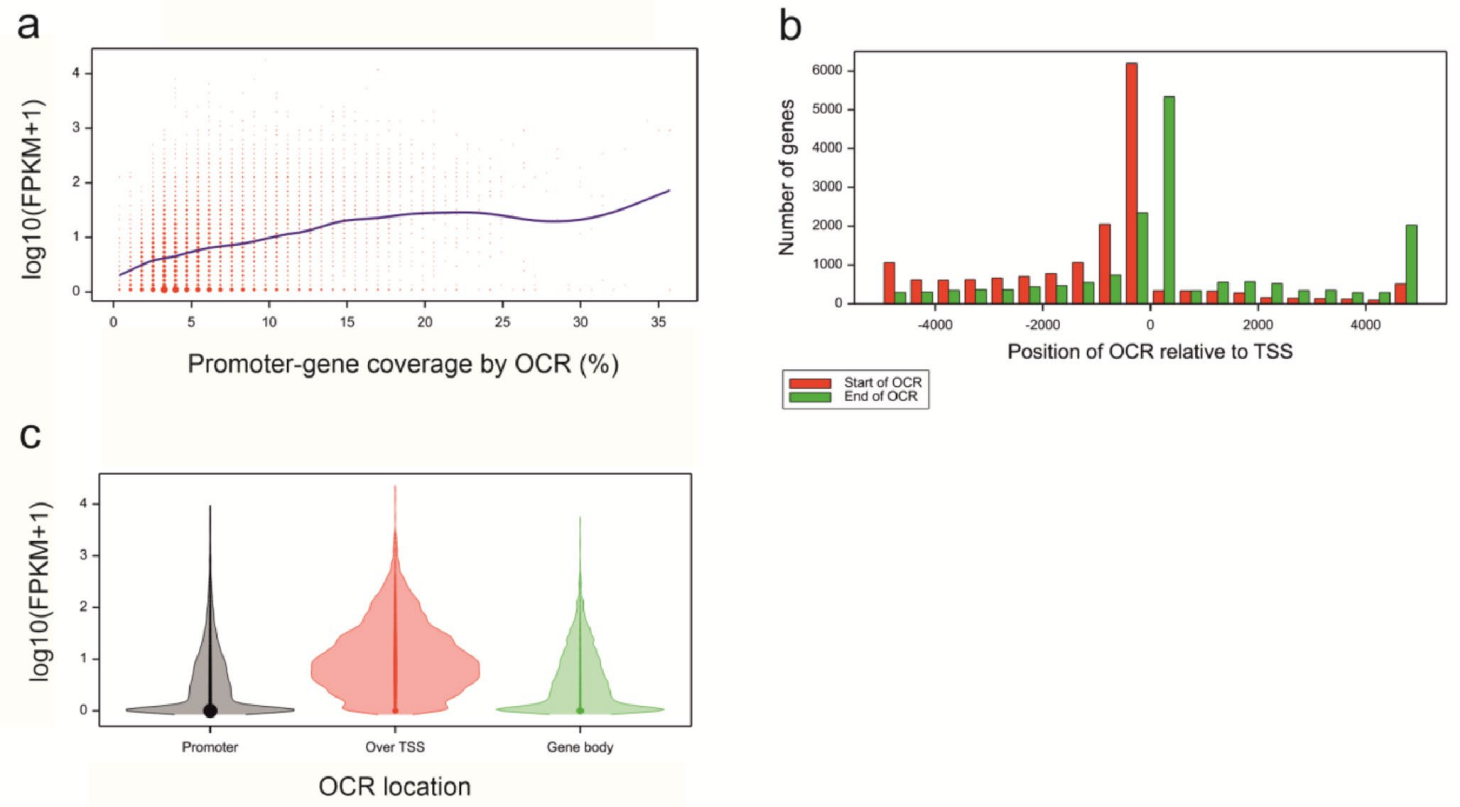
**(a)** Gene expression (FPKM) v. percentage of promoter-gene interval covered by OCR. **(b)** Histogram of positions of OCRs relative to TSS of genes. **(c)** Distribution of gene expression levels in classes of genes with OCRs in promoter (6,237 genes), over TSS (8,140 genes), and in gene body (2,493 genes)

Within the subset of genes with OCRs in at least one replicate (16,870 genes), there was an indication that the mean expression increased with increasing coverage of the promoter-gene interval by OCR (Fig. 7a). The dominant position of the OCR start relative to transcription start site (TSS) was in the interval [-500 nt, 0 nt], and that of the OCR end - in the interval [0 nt, 500 nt], in the core promoter and just downstream of the TSS (Fig. 7b). Gene expression was substantially higher in the group of genes with access to the core promoter and TSS than that in the group of genes with OCRs in more distal promoter regions or in the gene body (Fig. 7c).

**Fig. 8:**
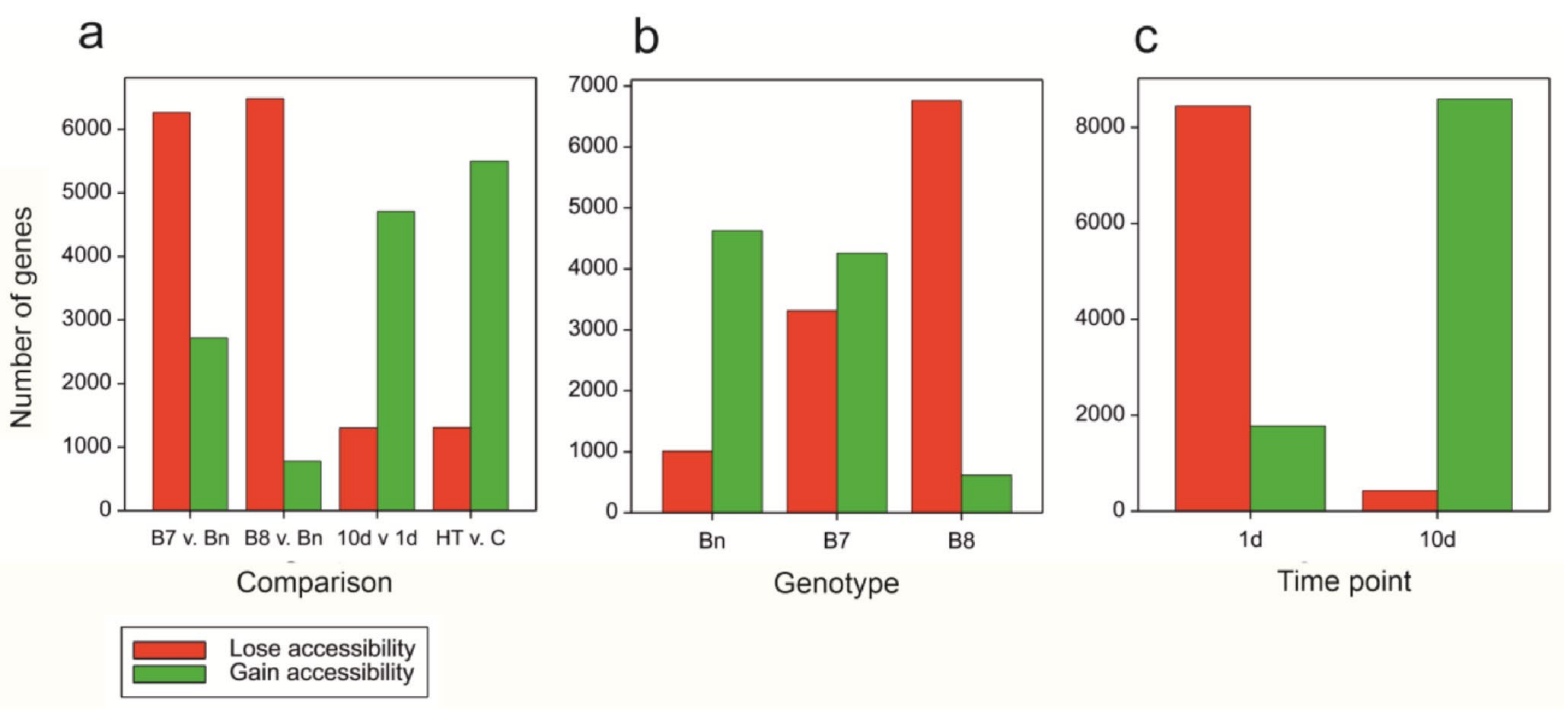
Numbers of genes changing the accessibility status between: **(a)** Genotypes, time points and temperature variants, **(b)** Temperature variants HT and C for three genotypes, **(c)** Temperature variants HT and C for time points 1d and 10d

To assess the relationship between change of accessibility and change of expression, genes changing accessibility status between experimental variants were found (“lose accessibility”: changing at least one OCR to none; “gain accessibility”: changing from none to at least one OCR) (Fig. 8). To some extent, the number of genes with changing status is a consequence of the different numbers of OCRs and genes having OCRs for different variants (the imbalance of marginal OCR sets and gene sets shown above), especially for HT v. C comparisons. However, the largest relative number of genes gaining accessibility under heat to number of genes losing accessibility was clearly observed at 10d, when the action of the high temperature was prolonged (Fig. 8c).

Genes losing OCRs between variants were more likely to decrease than to increase their expression levels in the following comparisons: B7 v. Bn, HT v. C, and HT vs. C for all genotypes and for 1d (Figure S8). However, this was not true for increased accessibility and increased expression levels.

The set of 13 genes with decreased accessibility and expression in comparison to B7 v. Bn contained, as expected, eight genes located in the deletion region neighboring the gene *sdw1* (3HG0307130; Mikołajczak et al. 2022). The other five were a cluster of 2HG0098640, 2HG0098890, and 2HG0098930 located on chromosome 2 H, and another cluster of 6HG0623240 and 6HG0624190 located on chromosome 6 H. Most of them encoded proteins whose function has not yet been defined, while some encoded proteins involved in signal transduction, including serine/threonine kinase (2HG0098890), kinase domain-containing protein (2HG0098930), serine incorporator (6HG0623240), NB-ARC domain-containing protein (2HG0098640), and Fe2OG dioxygenase domain-containing protein (3HG0307130).

**Fig. 9:**
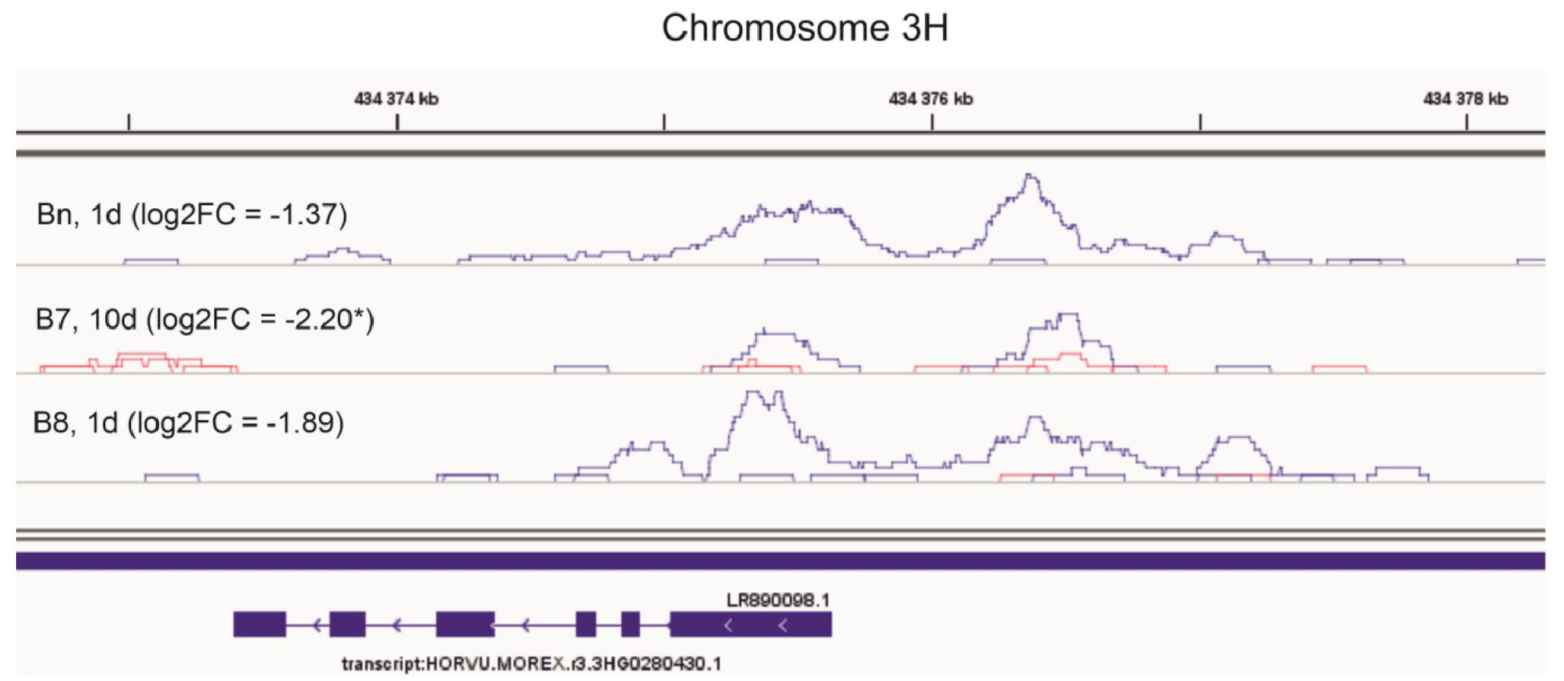
Profiles of enrichment in ATAC-seq NGS reads in the promoter-gene region of 3HG0280430 gene. Blue - replicates under control temperature (C) suggesting an OCR, red - replicates under high temperature (HT) suggesting no OCR; log2FC values for a decrease of gene expression under HT v. C in parentheses, * for a significant comparison

The set of 45 genes which showed reduced accessibility and expression in comparison HT v. C included genes encoding proteins involved in the hormone signal transduction (2HG0190070, 2HG0193430, 2HG0201500), regulation of nitrogen compound metabolic process (3HG0235850), regulation of transcription (4HG0333220), protein processing (5HG0430240), biosynthesis of cutin, suberine and wax (3HG0280430; Fig. 9), carotenoid (4HG0400960), secologanin and strictosidine (5HG0477240), and sucrose (7HG0667840). Of particular interest was the gene encoding HSFA6B, a protein involved in the HSFA7/HSFA6B-regulatory network (4HG0348370), which connects ABA signaling and ABA-mediated heat responses.

The sets of 22, 158, and 215 genes that showed decreased accessibility and expression in comparison to HT v. C for Bn, B7, and B8, respectively, contained genes whose protein products possessed DNA-binding transcription factor activity and were engaged in the regulation of transcription, e.g. 3HG0235850; 4HG0411810 for Bn, 2HG0170820, 3HG0245960, 3HG0304380, and 5HG0463370 for B7; and 2HG0196510, 3HG0278140, 4HG0379660, 6HG0628110, and 7HG0710560 for B8. In addition, genes involved in plant metabolic processes, hormone signal transduction, and the MAPK signaling pathway were identified in all genotypes. Moreover, the heat shock protein DnaJ (5HG0461670) was identified only in B7, whereas the heat shock factor (HSF)-type was identified in both the B7 (2HG0187550, 5HG0497990) and B8 (7HG0686980, 5HG0497990, 5HG0486440) genotypes. In Bn and B8 we also identified genes encoding the HSFA6B protein involved in the HSFA7/HSFA6B-regulatory network (4HG0348370 for Bn and 5HG0517140 for B8). Interestingly, the two genes involved in the GA-mediated signaling pathway were found only in B7. For one of these comparisons, for B8, owing to the large number of genes (215), overrepresented GO terms were found, e.g., those related to processes of regulation and transcription factor activity (Table S5).

For the set of 1,007 genes with decreased accessibility and expression in comparison of HT v. C for 1d, overrepresented GO terms were found, e.g., those related to processes of regulation and transcription factor activity, represented by genes distributed in all chromosomes, with special attention to six genes encoding heat shock factors (HSPs) localized in chromosomes 2 H (2HG0134940), 3 H (3HG0286890, 3HG0305080), 4 H (4HG0417910), and 5 H (5HG0497990, 5HG0486440). In this set, there were also genes whose protein products were involved in heat stress tolerance, including ubiquitin ligases (1HG0075150, 6HG0603650, 6HG0628340, 6HG0628400, 7HG0668940) and ion transporters (3HG0298980, 5HG0420230, 5HG0513990, 6HG0605270), as well as regulatory proteins, such as RLKs and MAPKs (1HG0005040, 1HG0015720, 1HG0079530, 2HG0112440) (Tables S1 and S4).

### Targeted analysis of genes related to “heat” and “gibberellin”

Based on the annotation, 72 and 32 genes were found with some roles in reaction to heat and in GA biosynthesis or signaling, respectively (these genes are identified in columns “heat” and “gibberellin” in Table S6).

Among genes related to “heat,” there were 11 DEGs in at least one of HT v. C comparison. Most of these genes were upregulated (8). Among these genes, eight changed accessibility in at least one comparison, HT v. C, in most cases, by losing OCRs, especially in the B8 genotype. However, in general, modifications in chromatin accessibility were not concordant with transcriptional changes. In parallel, this gene loses its chromatin accessibility in B8 cells under heat stress. There were seven genes with decreased chromatin accessibility in both *sdw1* NILs under heat but the stress-induced downregulation was found only in one case, namely 5HG0486440 (annotated to salicylic acid signaling according to Plant Reactome database) in B8. Other genes whose expression was altered in response to heat treatment were heat shock protein 21-like (6HG0578520), heat shock factor (HSF)-type (1HG0082040, 1HG0083950, 4HG0402170), heat intolerant 4 protein (2HG0201910), heat shock protein 70 family (4HG0387610, 4HG0387870, 5HG0441860), and heat shock protein DnaJ (4HG0395600, 5HG0454880, 5HG0456050).

Within 32 genes related to “gibberellin” there were six DEGs in at least one of HT v. C comparisons; all of them were downregulated. Most showed lost chromatin accessibility in *sdw1* NILs, whereas in Bn, only two genes changed (gained) accessibility under heat (Table S6). Interestingly, none of the GA-related genes showed altered expression in Bn in response to heat treatment, whereas three stress-induced DEGs were found for B7, and one of them was shared with B8 (HT v. C). Notably, the reduced expression of DEGs in B7 corresponded to lost chromatin accessibility, including two genes involved in GA gibberellic acid mediated signaling pathway, namely 2HG0158690 (annotated to WRKY transcription factor) and 4HG0399750 (annotated to F-box protein SNE), as well as *GA2ox3* involved in the regulation of GA biosynthesis.

Of special interest were two genes involved in the GA biosynthesis, namely, *GA20ox2* (3HG0307130), *GA20ox3* (3HG0306160), and a gene *GA2ox3* (3HG0289810) whose protein product participates in degradation of bioactive gibberellin in plants leading to dwarfing phenotype, as well as two genes involved in GA signaling annotated to the soluble GA receptor (1HG0062680) and DELLA repressor protein (4HG0336620). Among them, only in two cases, the changed accessibility of chromatin corresponded to modified expression of the gene in response to HT, namely *GA2ox3* (mentioned above) and 1HG0062680, for which the loss of chromatin accessibility in response to early stress was related to the downregulation of the gene, whereas the gained accessibility under prolonged stress did not affect gene expression (Table S2). In turn, *sdw1* (3HG0307130) had OCRs in Bn and B8, at 1d under C, and in Bn at 10d under HT. Therefore, it lost accessibility in the comparison HT v. C at 1d but gained at 10d. This gene was not a DEG in any comparison, except for B7 v. Bn (due to the complete deletion of *sdw1* in B7).

## Discussion

Long-range control of gene transcription by distal *cis*-regulatory elements (CREs) is an essential and often studied characteristic of plant genomes. To date, many fundamental aspects of distal CREs, including their prevalence, evolution, chromatin signatures, and mechanisms of action, remain unclear. In this study, we used ATAC-seq profiling of accessible chromatin to investigate the transcriptional regulatory landscape of the barley genome. The similarities and differences in accessible chromatin regions in three barley genotypes carrying different allelic forms of the *sdw1* gene encoding GA20ox subjected to high temperatures were analyzed. We adopted the Fluorescence-Activated Nuclei Sorting (FANS) method to purify barley crown tissue nuclei. We also investigated the correlation between different types of OCRs and gene expression. Finally, to identify the regulatory networks that affect the regulation of gene expression (promotion or repression), we integrated ATAC-seq and RNA-seq data generated in the same context.

The datasets obtained by applying the ATAC-seq protocol were unbalanced in the sense that the number of reads mapped in the genome was different for different combinations of levels of the studied factors (genotypes, temperature regime, and time). This could be caused by a different number of amplification cycles necessary for obtaining the DNA quantities required for sequencing (from 9 to 19). We reduced the effects of this imbalance by subsampling the reads; however, the resulting numbers of OCRs (peaks) differed. This prevented us from analyzing chromatin accessibility at the factor combination level. Instead, we performed a marginal analysis of factor levels, which revealed the main differences between barley forms and time points and the effects of heat treatment. The disadvantage of this analysis is the lack of inference regarding the interaction of the experimental factors and, in consequence, the fact, that differences between levels of a factor (say, temperature) may occur at different levels of other factors (genotype or time point). Regarding the observed imbalance, we also showed that it was caused mainly by non-genic OCRs, which were less repeatable than the genic or promoter ones. The possible non-optimalities of the laboratory procedures did not affect much our inference on the accessibility of genic and promoter regions and their functional classifications. A broad validation of the OCRs found by us against other results is not possible due to a lack of published data; however, our data confirmed the existence of OCRs in two regions of chromosome 5 presented recently by Pavlu et al. (2024) (Supplementary Figure S9).

Our data revealed that OCRs were widely distributed across all chromosomes of the barley genome; however, the hypersensitive chromatin regions occurring within the genes were repeated more frequently than those outside the gene intervals. In a comparison of open chromatin among the various genotypes and factor levels, we found that the genomic frequency of ATAC-seq peaks was rather similar in the transcribed regions and promoters, whereas the differences were caused mainly by the presence of numerous OCRs located outside the promoter-gene regions. This was observed mainly in genotype B7 at high temperatures (HT). Similar findings were reported by Maher et al. (2018), who noticed that approximately 70–80% of the transposase hypersensitive sites (THSs/peaks) were found outside of transcribed regions in *A. thaliana*,* M. truncatula*, *S. lycopersicum*, and *O. sativa* genomes and that these THSs showed marked differences between the species studied. Furthermore, we found agreement between the number of open chromatin regions and the number of accessible genes in the barley genome. The largest number of open chromatin regions and corresponding genes was observed under longer heat stress (10d). Based on these data, we conclude that prolonged action at HT increases the accessibility of chromatin and genes. Dynamic changes in gene regulation often occur in response to environmental conditions (Zhang et al., 2022). The frequency of regions with variable accessibility varies widely depending on the specific stress factor (Raxwal et al. 2020). Zeng et al. (2019) identified multiple regions of increased availability that were frequently associated with differential gene expression in potato tubers after cold stress treatment. Moreover, Liang et al. (2021) recognized global reorganization of the 3D chromatin architecture of rice genomes during heat stress through a combined analysis of three-dimensional spatial organization and chromatin accessibility. This observation is consistent with recent studies in which heat stress induced rapid changes in heterochromatin organization and thus its accessibility, as well as dynamic interactions between promoters and distal regulatory elements in Arabidopsis, maize, and tomato (Sun et al. 2020; Huang et al. 2023). In contrast, Myers et al. (2023) revealed limited variability in chromatin accessibility in maize and Setaria in response to heat stress.

Through GO enrichment analysis of the genes present in differentially accessible chromatin regions for the three barley genotypes, two temperature regimes, and two time points, we observed enrichment in a set of genes that were involved in a large variety of biological processes. We focused our attention mainly on the largest set of genes with OCRs obtained at prolonged high temperatures, including calcium-dependent protein kinase, mitogen-activated protein kinase MAPK3, RLK, TIFY domain-containing transcriptional regulator, bZIP transcription factor, and regulatory protein NPR1. Numerous studies have indicated that these proteins are involved in plant responses to environmental constraints. For example, MAPK cascades participate in the response of plants to diverse environmental stresses, such as drought, salt, cold, and heat stress (Lin et al. 2021). Wu et al. (2015) showed that overexpression of *ZmMPK1* in Arabidopsis enhanced tolerance to heat stress by increasing proline content and decreasing MDA content. Other studies have revealed that *SlMAPK3* is a negative regulator of thermotolerance in tomatoes. Moreover, the antioxidant enzymes and *HSPs*/*HSFs* genes expression can be involved in *SlMAPK3*-mediated heat stress response (Yu et al. 2019). In contrast, functional analysis of *TIFY* family genes in *Fagopyrum tataricum* revealed that most *FtTIFYs* respond to various abiotic stresses, including high temperatures (Zhao et al. 2023).

Previous studies have suggested that crown tissue plays an essential role in plant performance under high-temperature stress (Prerostova et al. 2022). Our RNA-sequencing analyses, reported here and by Mikołajczak et al. (2022), based on the same RNA-seq raw data but different reference genome versions and sets of comparisons, provide novel information on gene expression in the crowns of three barley genotypes subjected to elevated temperatures and observed at two time points during stress. As expected, the effect of genotype on gene expression was weaker than that of temperature because genetically related but polymorphic forms of barley were used in this study. Despite the observed phenotypic differences between the Bowman and NIL lines, reflected by variations in active GA content, there were no significant changes in the GA biosynthetic genes expression level including *HvGA20ox2* and *HvGA20ox3* between genotypes under control conditions (Table S6). One reason may be the lack of polymorphism within the *HvGA20ox2* promoter between the Bn and B8 (data not shown), suggesting a similar mechanism of transcriptional regulation of the *HvGA20ox2* gene. On the other hand, a decrease of *SNE* gene expression encoding the F-box protein involved in DELLA degradation was noticed in the B7 line compared to the Bn genotype. This observation explains the increase in chromatin accessibility for the gene encoding DELLA protein in the B7 line which acts as a negative regulator of gibberellin (GA) signaling. It is also interesting to note the decreased expression of the *GA2ox3* gene encoding a key catabolic enzyme in deactivating GA in both NIL lines compared to the Bn genotype. Inactivation of GA catabolic enzymes abolishes the GA deactivation pathway, which can cause GA content to rise, thereby preventing the development of *dwarf* genotypes. Recently, Cheng et al. (2024) identified ten genes encoding *Gibberellin 2-oxidase* in the barley cultivated genotype Morex genome. They revealed that *GA2ox* genes are transcriptionally expressed in a tissue-specific way and implied divergent biological functions for each *GA2ox*. It was demonstrated that HvGA2ox4 can control plant height, the other HvGA2ox7 regulates seed growth and affects grain size and quality while one naturally occurring HvGA2ox8a haplotype was associated with decreased plant height, early flowering and wider and heavier seed (Cheng et al. 2024). This may explain the downregulation of *GA2ox3* gene expression in *semi-dwarf sdw1.a* and *sdw1.d* mutants and suggest that the GA2ox3 protein is not involved in the processes controlling plant height. Few DEGs were found in the genotype and time-point comparisons. The number of DEGs between B7 and Bn was similar to that between B8 and Bn, with half of the DEGs in common. Generally, the transcriptomic response to HT was similar for Bn and B8 but more intense for the B7 genotype, where we could identify a two-fold higher number of genes whose transcription patterns were responsive to temperature conditions. We also noticed that most DEGs were found for the HT v. C contrast at time point 1d (thrice more DEGs than those at the time point 10d) of which twice as many were downregulated. This is consistent with existing data, indicating that heat stress causes the downregulation of several genes related to various pathways. Poidevin et al. (2020) revealed that heat stress caused a dramatic downregulation of genes encoding membrane transporters, such as K^+^ and carbohydrate co-transporters, related to the germinated pollen of *A. thaliana.* Furthermore, transcriptomic data have shown dramatic changes in DEGs, with numerous genes being downregulated after 12 and 24 h of heat stress in sweet corn (Wang et al. 2023). In general, plants can respond to heat stress by activating various metabolic pathways depending on the variety, genotype, and the frequency and intensity of elevated temperatures. Moreover, other authors have shown that the time of day and the circadian clock are essential factors determining the heat stress-responsive transcriptome in Arabidopsis (Blair et al. 2019).

Chromatin accessibility is required but not sufficient to activate gene expression, which is also determined by the availability of recruited transcription factors or histone modifications (Klemm et al. 2019). However, previous studies have reported a significant correlation between gene expression and ATAC-seq signals in terms of gene promoter accessibility. This is in agreement with our data, in which a relationship was found between the change in accessibility and change in expression between the experimental variants. By combining the results of the RNA-seq and ATAC-seq differential analyses, we identified genes with high-temperature-induced changes in chromatin accessibility associated with expression alterations. Notably, we noticed that genes that lost chromatin accessibility had reduced transcript levels in comparisons of B7 v. Bn, HT v. C, and HT v. C for all genotypes and for 1d. Transcriptome analysis indicated that the candidate downregulated genes were involved in plant photosynthesis, metabolic processes (mainly the synthesis of secondary metabolites), hormone signal transduction, MAPK cascade, Ca^2+^ signaling, regulation of transcription, heat stress response, heat shock protein DnaJ, HSF-type, and HSFA6B-regulatory network. The lack of chromatin openness was positively correlated with decreased expression levels of these genes. According to Liang et al. (2021), chromatin accessibility in rice cultivars is consistent with expression dynamics in response to heat stress.

Analysis targeted to GA-related genes revealed that in genotype B7, three GA-related DEGs reacted specifically to elevated temperatures compared with the other genotypes. Based on ATAC-seq, we suggest that the downregulation of these genes may result from the loss of chromatin accessibility in response to stress. Apparently, the GA disorders caused by the total deletion of the *sdw1* gene were partially compensated for by the altered activity of these B7-specific genes, as 3HG0307130 (*GA2ox3*) and 2HG0158690 (annotated to WRKY transcription factor) are negative regulators of GA activity; namely, they are responsible for the degradation of GA and inhibition of GA signaling, respectively (Salas Fernandez et al. 2009; Phukan et al. 2016). Therefore, reduced expression can positively influence GA homeostasis under heat stress conditions. Moreover, B7-specific DEGs annotated to the WRKY and F-box (4HG0399750) domains were involved in salicylic acid and brassinosteroid signaling (according to the Plant Reactome database), respectively. Thus, they are promising candidates for hormone tradeoffs in the response of barley to high temperatures.

## Electronic supplementary material

Below is the link to the electronic supplementary material.

Supplementary Material 1

Supplementary Material 2

Supplementary Material 3

Supplementary Material 4

Supplementary Material 5

Supplementary Material 6

Supplementary Material 7

## Data Availability

All data generated and analyzed in this study are included in the published article and its supplementary information files. Additionally, high-throughput sequencing data used in this paper are available in the ArrayExpress repository (https://www.ebi.ac.uk/arrayexpress), accession number E-MTAB-13600 for ATAC-seq data, and E-MTAB-13599 for RNA-seq data – access open after publication. The data from public open-access database Ensemble Plants (https://plants.ensembl.org) was used for raw data processing.
