# Supplementary material for: Heat stress causes chromatin accessibility and related gene expression changes in crown tissues of barley (Hordeum vulgare)
Source: Plant Mol Biol. 2024 Oct 22;114(6):115. doi: 10.1007/s11103-024-01509-x (PMC11496342; doi:10.1007/s11103-024-01509-x)
Supplement: Supplementary file 1 — Supplementary Material 1 [file 11103_2024_1509_MOESM1_ESM.doc]

| 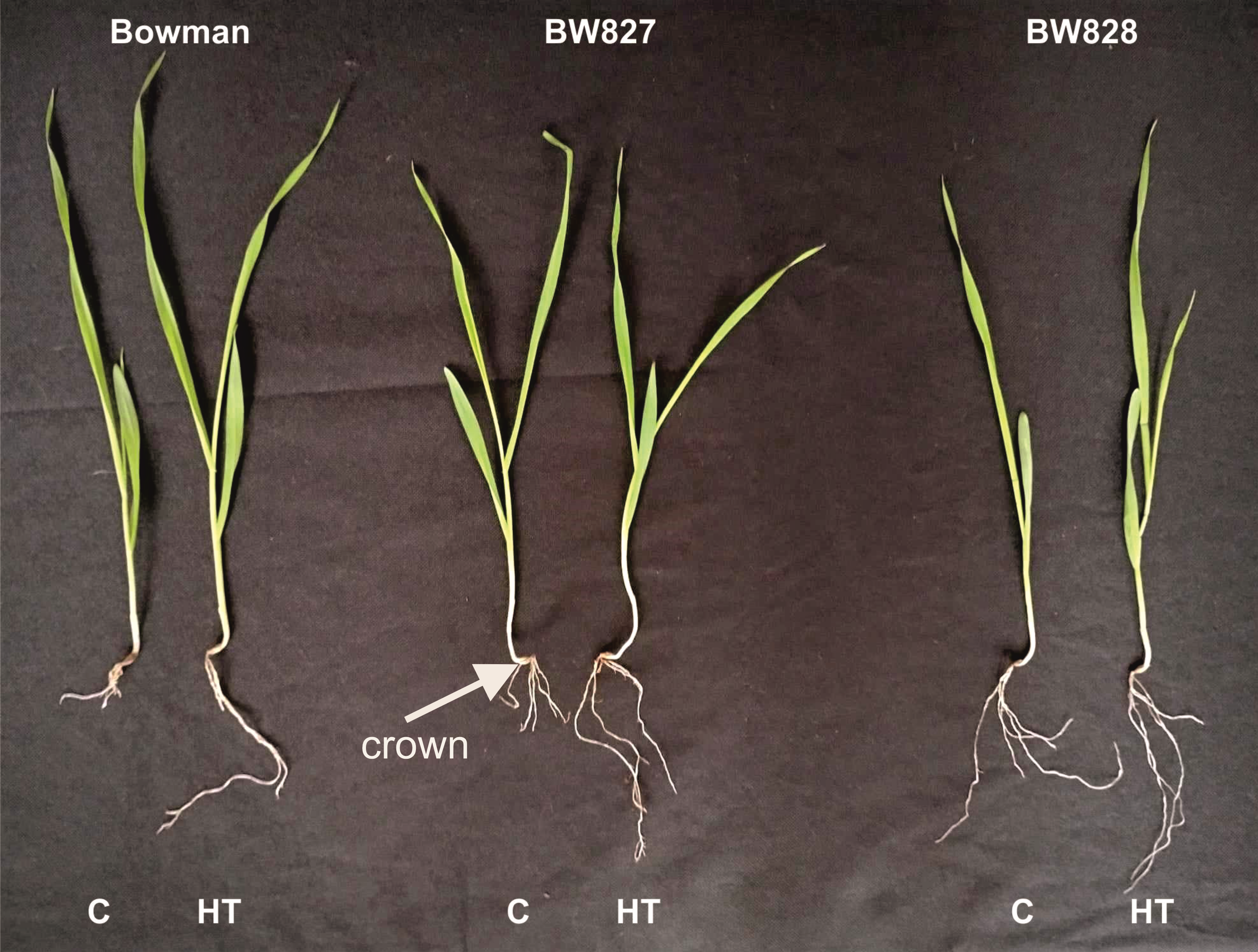 |
| --- |
| **Figure S1.** Crown tissue of three barley genotypes: Bowman (Bn), BW827 (B7) and BW828 (B8) under control (C) and high temperature conditions (HT) for 10 days. |

| A | B |
| --- | --- |
| | Number of peaks merged into OCR | % of OCRs intersecting with promoter-gene interval | | Total | | --- | --- | --- | --- | | no | yes | | 1-2 | 75.24 | 24.76 | 48069 | | 3-5 | 58.12 | 41.88 | 15545 | | 6-10 | 51.37 | 48.63 | 5856 | | 11-15 | 60.56 | 39.44 | 393 | | 15-54 | 55.04 | 44.96 | 238 | | Total | 69.3 | 30.7 | 70101 | | Pearson χ^2^ = 2632.84 with 4 d.f., P < 0.001 | | | | | | Number of peaks merged into OCR | % of OCRs intersecting with promoter | | Total | | --- | --- | --- | --- | | no | yes | | 1-2 | 30.81 | 69.19 | 11901 | | 3-5 | 23.73 | 76.27 | 6510 | | 6-10 | 18.01 | 81.99 | 2848 | | 11-15 | 14.19 | 85.81 | 155 | | 15-54 | 6.54 | 93.46 | 107 | | Total | 26.74 | 73.26 | 21521 | | Pearson χ^2^ = 276.31 with 4 d.f., P < 0.001 | | | | |
| 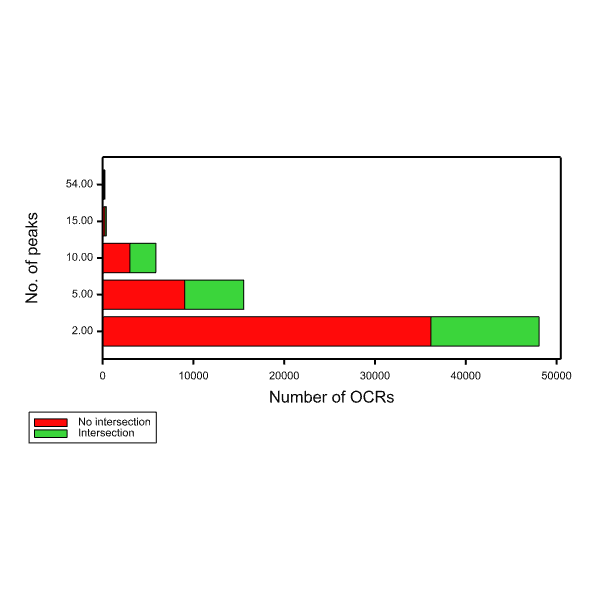 | 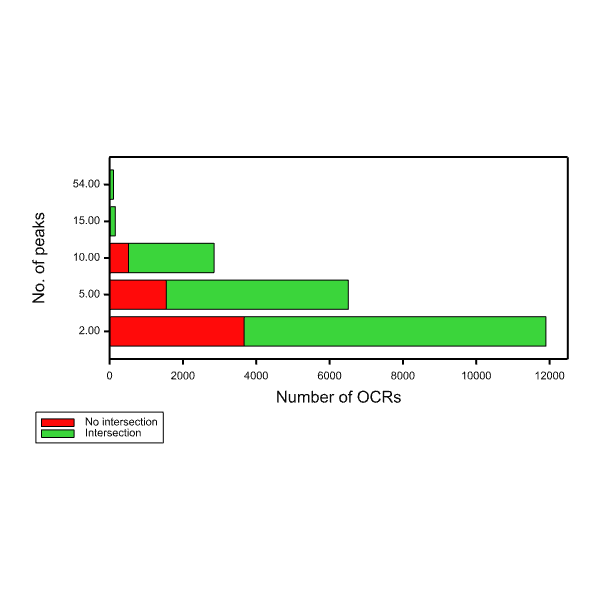 |

**Figure S2.** Relationships between frequency of occurrence of OCRs (number of peaks merged into OCR) and OCR position. A. Intersection of OCRs with promoter-gene intervals, B. Intersection of OCRs with promoters.

| **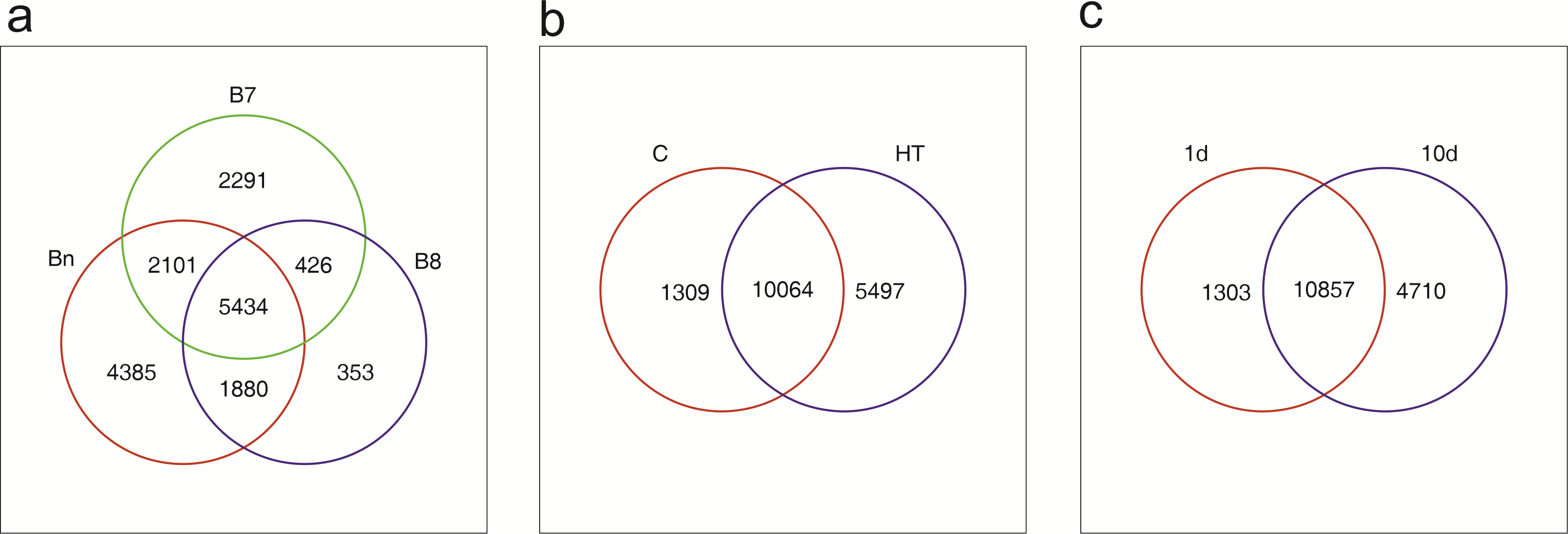** |
| --- |
| **Figure S3.** Common and specific genes having OCRs in the marginal sets corresponding to levels of factors: a. Genotype; b. Temperature; c. Time point. |
| **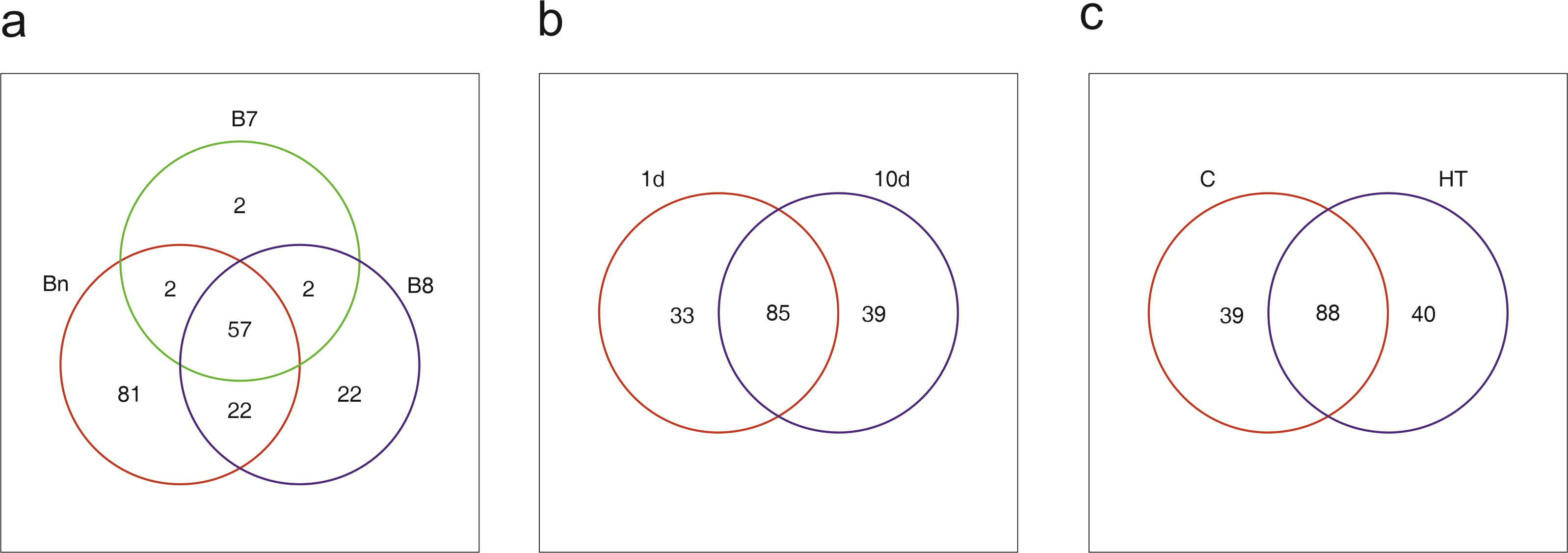** |
| **Figure S4.** Number of overrepresented GO terms common and specific for sets of genes with OCRs. a. Marginal sets for genotypes; b. Marginal sets for time points; c. Marginal sets for temperature regimes. |

**Figure S5.** Representative terms for GO terms highly overrepresented (FDR < 0.001) in the set of all genes with accessibility marks (Revigo tool, http://revigo.irb.hr/, set size = small, species = *Oryza japonica*, simil. measure = SimRel). A, Biological process; B, Molecular function; C, Cellular component.

| A |
| --- |
| 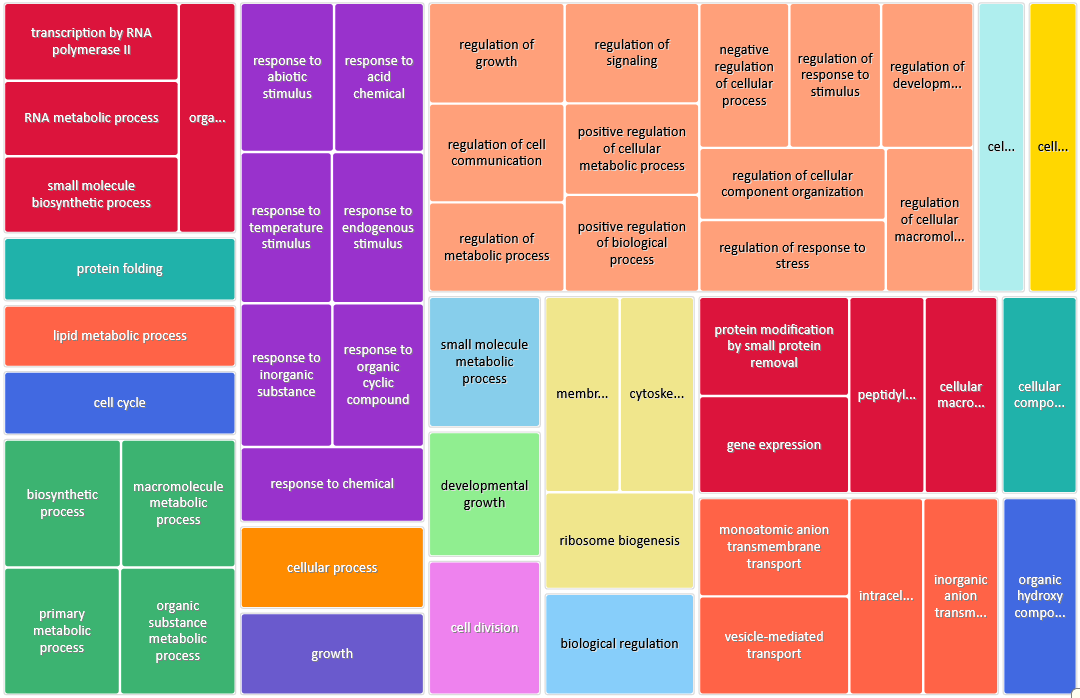 |
| B |
| 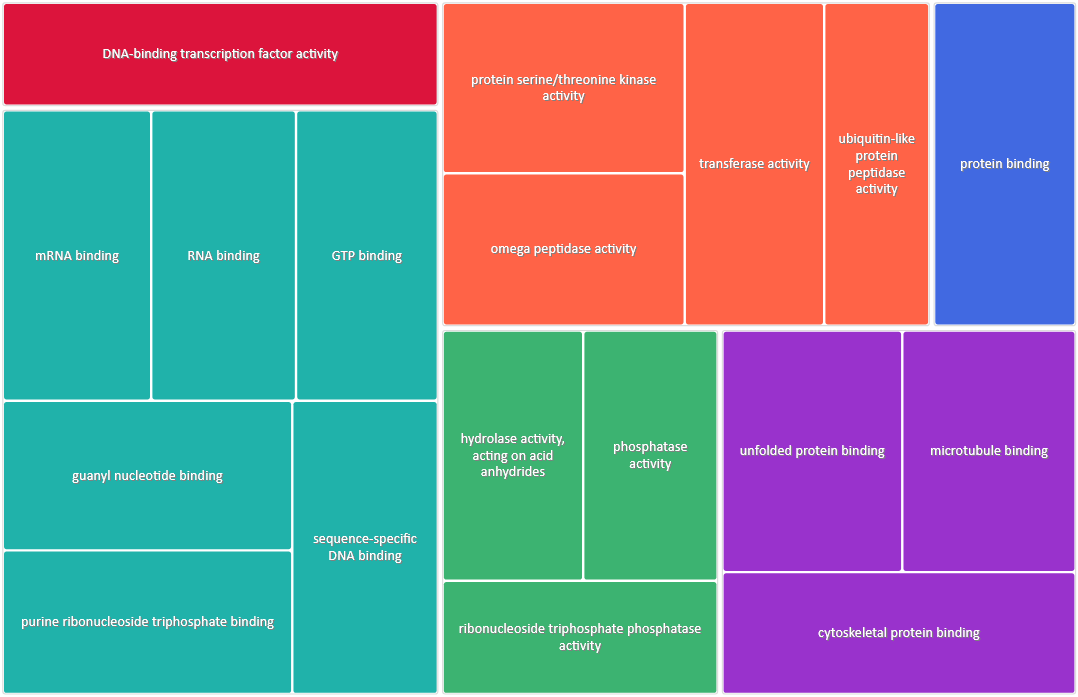 |

| C |
| --- |
| 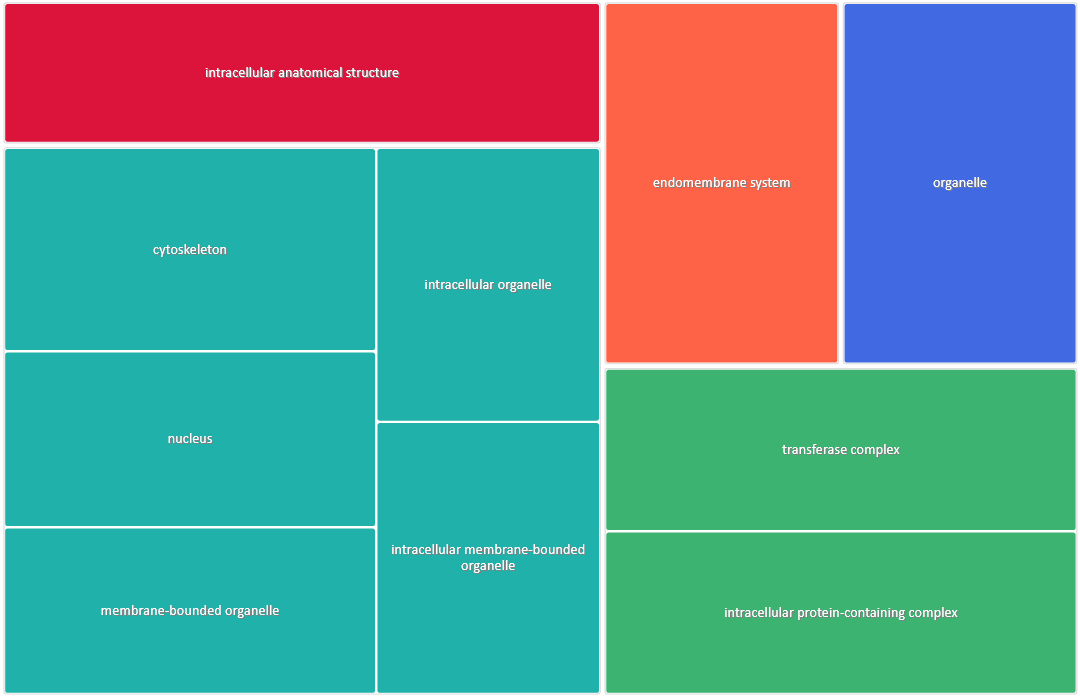 |

| **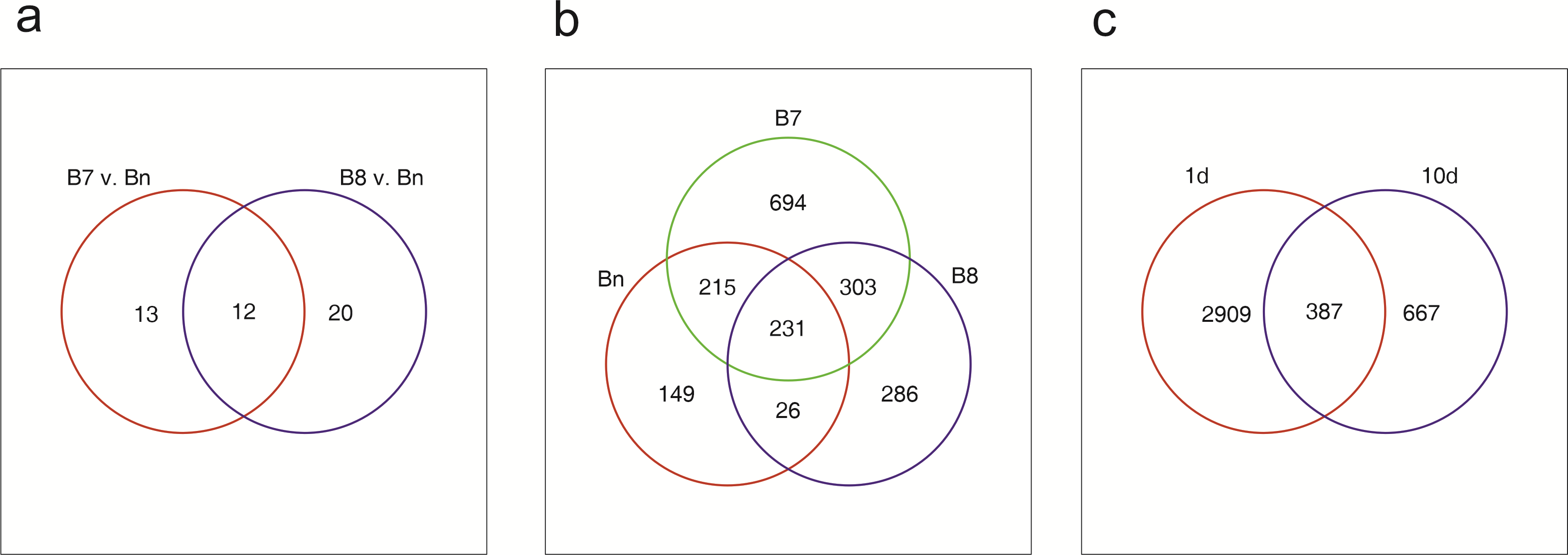** |
| --- |
| **Figure S6.** Number of DEGs common and specific for different comparisons. a. Comparison of NILs v. Bowman; b. Comparison of HT v. C for three genotypes; c. Comparison of HT v. C for two time points. |
| **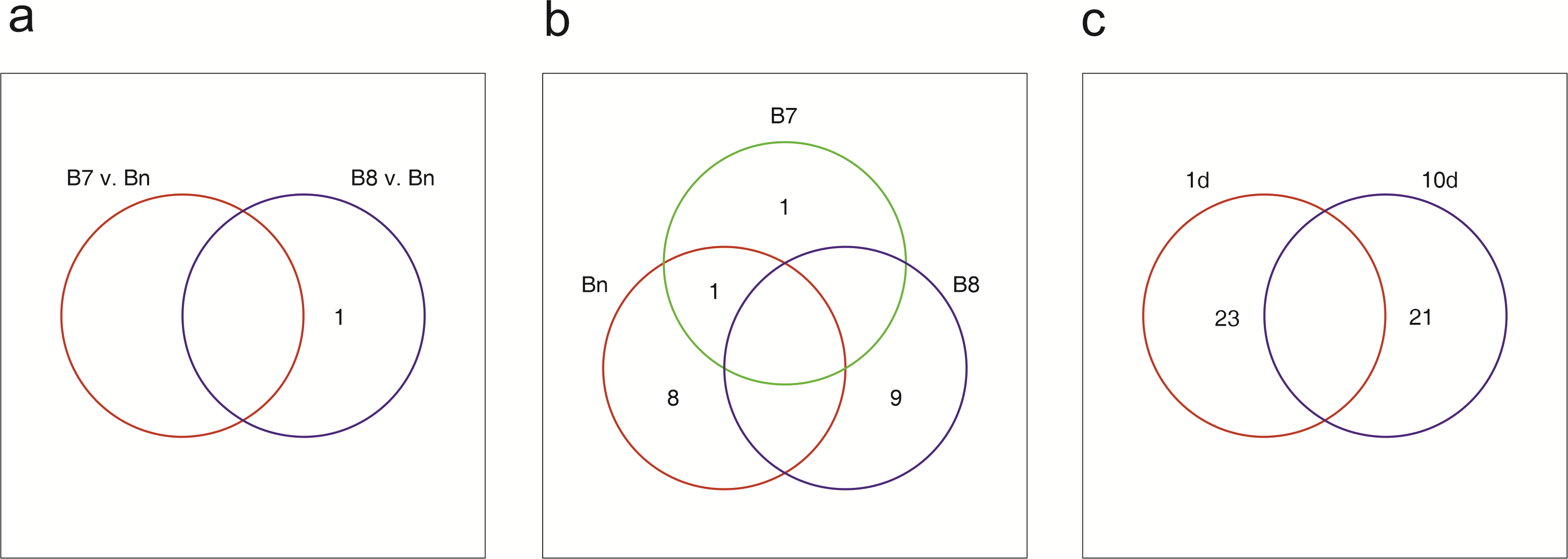** |
| **Figure S7.** Number of overrepresented GO terms common and specific for the sets of DEGs for comparisons of gene expression. a. Comparison of NILs v. Bowman; b. Comparison of HT v. C for three genotypes; c. Comparison of HT v. C for two time points. |

| 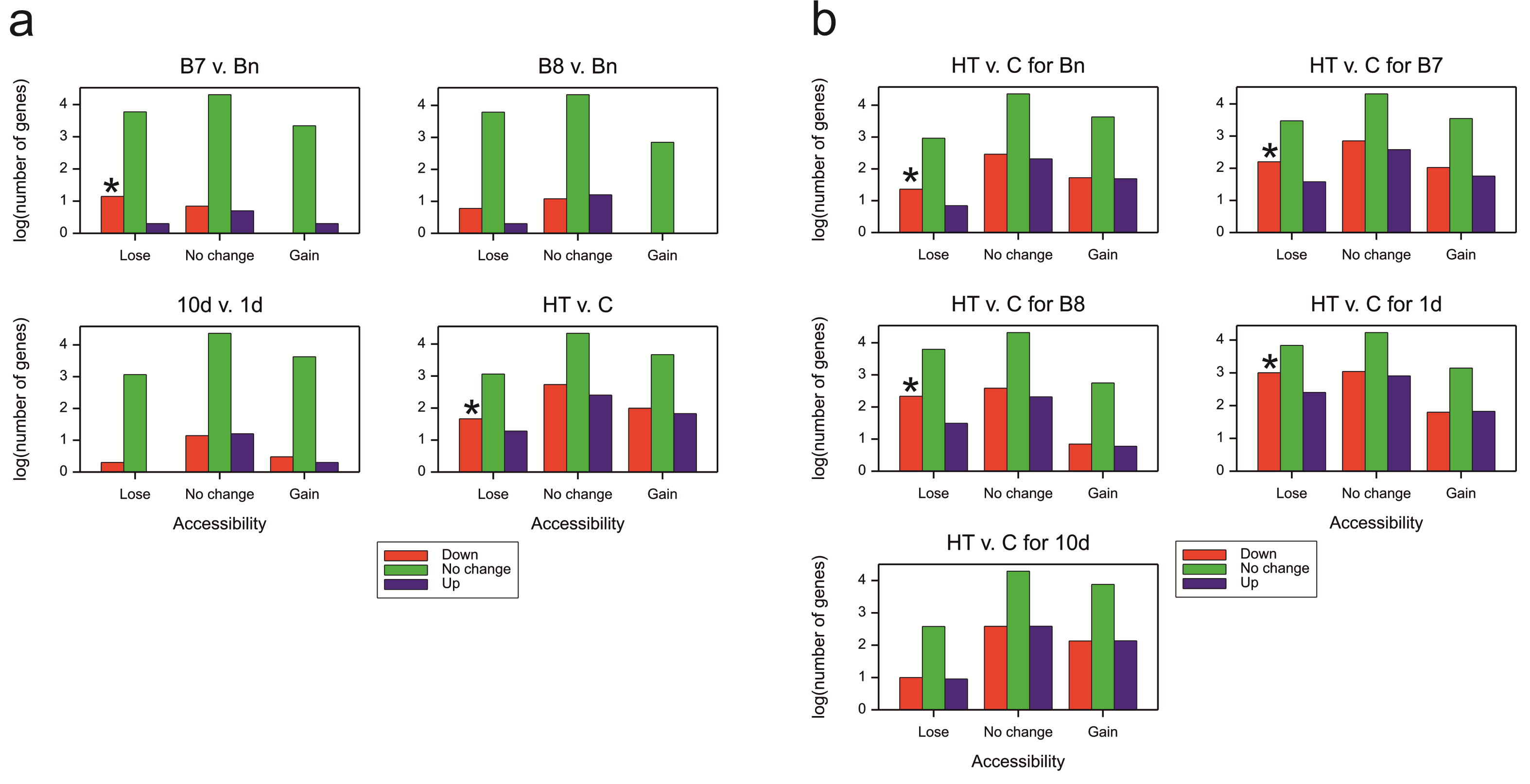 |
| --- |
| **Figure S8.** Results of testing homogeneity of distributions of genes down-, no- or upregulated within the groups losing, not changing, or gaining accessibility. Graphs show numbers of genes differentially regulated and differentially accessible in the comparisons: a) between experimental variants; b) between HT and C for different genotypes and time points. Overrepresented categories of genes (all downregulated) are marked by stars. Below are P values for testing homogeneity of distributions, cases of significance are in bold.  Comparison P value for homogeneity test  **B7 v. Bn < 0.001**  B8 v. Bn 0.406  10d v. 1d 0.734  **HT v. C 0.006**  **HT v. C for Bn 0.023**  **HT v C for B7 < 0.001**  **HT v C for B8 < 0.001**  **HT v C for 1d < 0.001**  HT v. C for 10d 0.623 |

| 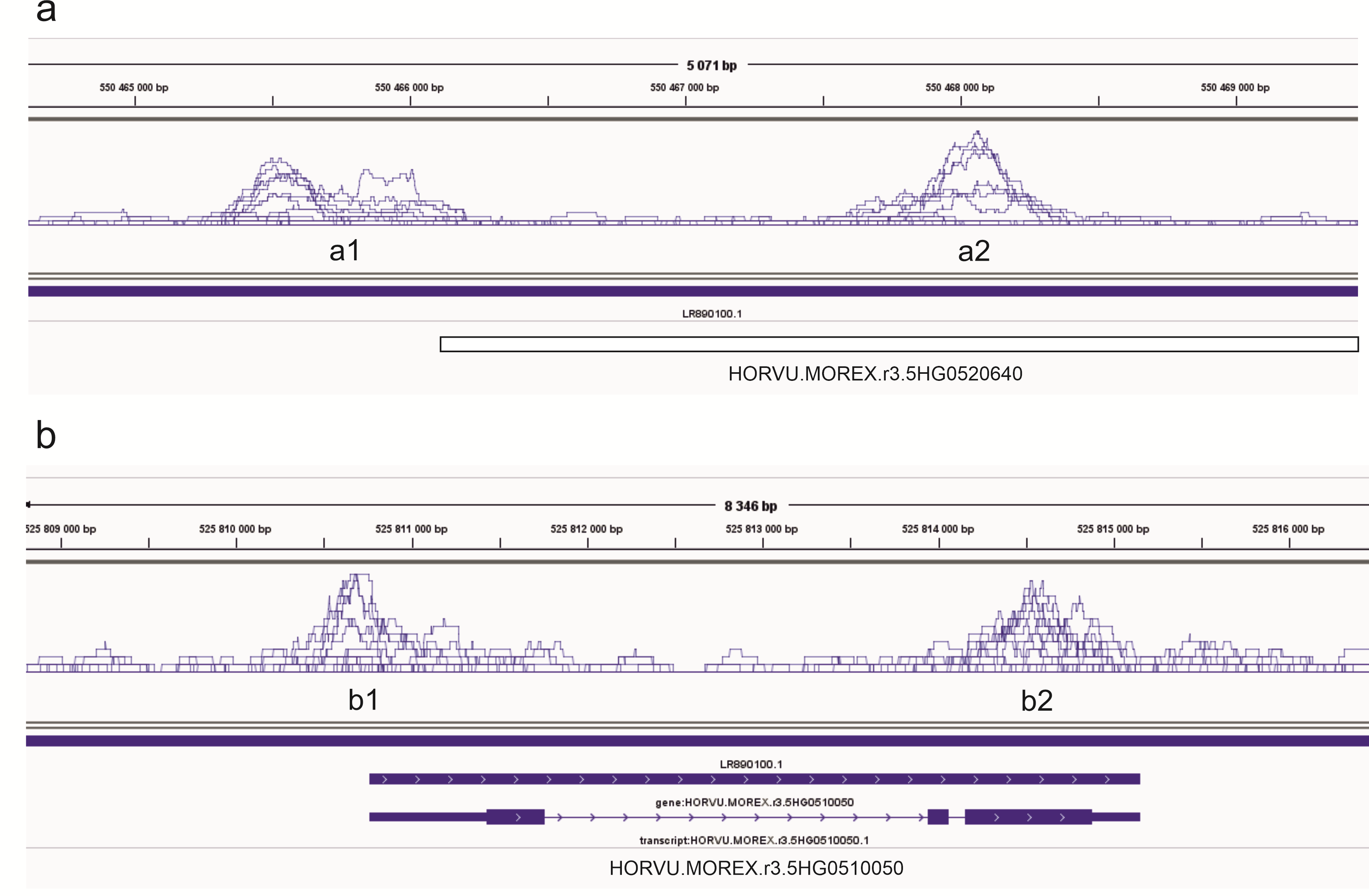 |
| --- |
| **Figure S9.** OCRs validated against results shown by Pavlu et al. (2024; Figure 7). a. OCRs found in chromosome 5, region 5H:550460000-550469000; OCRs a1 and a2 are located, respectively, in the promoter and first intron of 5HG0520640, which is shown only schematically as it is a low-confidence gene, not used in our data analysis. b. OCRs b1 and b2 found, respectively, in the promoter and exon of 5HG0510050. Profiles represent ATAC-seq read coverage in all samples. |
